# Preserving Neuronal Chemical Messengers: Heat Stabilization Versus Snap Freezing for Improved MALDI Mass Spectrometry Imaging of Brain Tissues

**DOI:** 10.1111/jnc.70122

**Published:** 2025-06-16

**Authors:** Emanuela Salviati, Dominika Luptáková, Anna Nilsson, Reza Shariatgorji, Pietro Campiglia, Nikita Tjernström, Erika Roman, Per E. Andrén

**Affiliations:** ^1^ Department of Pharmaceutical Biosciences, Spatial Mass Spectrometry, Science for Life Laboratory Uppsala University Uppsala Sweden; ^2^ Department of Pharmacy University of Salerno Fisciano SA Italy; ^3^ Institute of Microbiology of the Czech Academy of Sciences Prague Czech Republic; ^4^ Department of Pharmaceutical Biosciences, Neuropharmacology and Addiction Uppsala University Uppsala Sweden; ^5^ Department of Animal Biosciences, Division of Anatomy, Physiology, Immunology and Pathology Swedish University of Agricultural Sciences Uppsala Sweden

**Keywords:** brain, mass spectrometry imaging, metabolites, neuropeptide, neurotransmitter, postmortem degradation

## Abstract

One of the main challenges in analyzing chemical messengers in the brain is the optimization of tissue sampling and preparation protocols. Limiting postmortem time and terminating enzyme activity is critical to identify low‐abundance neurotransmitters and neuropeptides. Here, we used a rapid and uniform conductive heat transfer stabilization method that was compared with a conventional fresh freezing protocol. Together with a selective chemical derivatization method and an optimized quantitation approach using deuterated internal standards, we spatially mapped neurotransmitters and their related metabolites by matrix‐assisted laser desorption/ionization mass spectrometry imaging (MALDI‐MSI) in rat brain tissue sections. Although the heat stabilization did not show differences in the levels of dopamine, norepinephrine, and serotonin, their related metabolites 3,4‐dihydroxyphenylacetaldehyde, 3,4‐dihydroxyphenylacetic acid, homovanillic acid, 3‐methoxy‐4‐hydroxyphenylacetaldehyde, dihydroxyphenylethyleneglycol, and 5‐hydroxyindoleacetic acid were all significantly lower, indicating reduced neurotransmitter postmortem turnover ratios. Heat stabilization enabled detection of an increased number and higher levels of prodynorphin, proenkephalin, and tachykinin‐derived bioactive neuropeptides. The low‐abundant C‐terminal flanking peptide, neuropeptide‐γ, and nociceptin remained intact and were exclusively imaged in heat‐stabilized brains. Without heat stabilization, degradation fragments of full‐length peptides occurred in the fresh frozen tissues. The sample preparation protocols were furthermore tested on rat brains affected by acute anesthesia induced by isoflurane and medetomidine, showing comparable results to non‐anesthetized animals on the neurotransmitters level without significant changes. Our data provide evidence for the potential use of heat stabilization prior to MALDI‐MSI analyses to improve the examination of the in vivo state of neuronal chemical messengers in brain tissues not impacted by prior acute anesthesia.

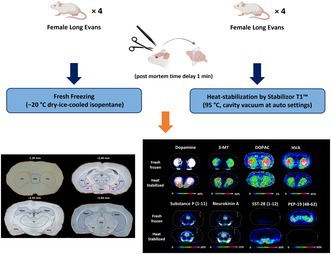

## Graphical abstract

Rapid heat stabilization of brain tissue preserves the natural levels and spatial distribution of neurotransmitters and neuropeptides better than traditional snap freezing. Using matrix‐assisted laser desorption/ionization mass spectrometry imaging (MALDI‐MSI), we show that heat stabilization effectively prevents postmortem enzymatic degradation, enabling more accurate visualization of the brain's chemical messengers. Our results demonstrate that this method not only improves the detection of intact neuropeptides but also reduces metabolite accumulation, offering a more accurate representation of the in vivo neurochemical state. This approach enhances the reliability of brain molecular imaging, particularly for studying neurological diseases and treatment effects.

**Abbreviations**
3‐MT: 3‐Methoxytyramine
5‐HIAA: 5‐hydroxyindoleacetic acid
5‐HIAL: 5‐hydroxyindolealdehyde
5‐HT: 5‐hydroxytryptamine (serotonin)
ACN: acetonitrile
DA: dopamine
DHB: 2,5‐dihydroxybenzoic acid
DOPAC: dihydroxyphenylacetic acid
DOPAL: 3,4‐dihydroxyphenylacetaldehyde
DOPEG: dihydroxyphenylethylene glycol
DynA: dynorphin A
FF: fresh frozen
FMP‐10: 4‐(anthracen‐9‐yl)‐2‐fluoro‐1‐methylpyridin‐1‐ium iodide salt
FMW: focused microwave irradiation
FTICR: fourier‐transform ion cyclotron resonance
GABA: γ‐aminobutyric acid
HS: heat stabilized
HVA: homovanillic acid
IS: internal standards
ITO: indium tin oxide
LC: liquid chromatography
MALDI‐MSI: matrix‐assisted laser desorption/ionization mass spectrometry imaging
MCH: melanin concentrating hormone
MOPAL: 3‐methoxy‐4‐hydroxyphenylacetaldehyde
MS: mass spectrometry
MS/MS: tandem MS
NE: norepinephrine
N‐EI: neuropeptide EI (neuropeptide glutamic acid‐isoleucine)
PC1: prohormone convertase 1
PCA: principal component analysis
PDYN: prodynorphin
PENK: proenkephalin
PEP‐19: 6.7 kDa neuronal calmodulin‐binding polypeptide
Q‐TOF: quadrupole time‐of‐flight
RMS: root‐mean square
RRID: research resource identifier
SP: substance P
SST: somatostatin
α‐Neo: α‐neoendorphin
β‐Neo: β‐neoendorphin

## 1 Introduction

In the central nervous system, many different endogenous substances function as chemical mediators, facilitating neuronal activity (Greengard 2001; Kandel et al. 2021). Neurotransmitters and neuropeptides mediate the synaptic transmission between neuronal cells in the brain (Kandel et al. 2021). They are inactivated by highly specific transporter systems or enzymes, and depending on the mode of inactivation, these molecules are considered true neurotransmitters or neuromodulators (Russo 2017). Changes in the concentration of neurotransmitters and neuropeptides are involved in several neurological diseases such as Parkinson's disease, Alzheimer's disease, depression, and substance use disorders (Bartus et al. 1982; Hulme et al. 2020; Lotharius and Brundin 2002; Marin et al. 2015; Politis and Niccolini 2015; Sgroi et al. 2016; Skold et al. 2006). Furthermore, administration of drugs has the capacity to modify the concentration of signaling molecules (Baijnath et al. 2022). In addition to measuring concentrations of the neuromodulatory compounds in brain tissues, quantitative imaging of the spatial distribution of multiple signaling molecules in the brain is of great importance (Hulme et al. 2020; Shariatgorji et al. 2019; Shiaratgorji et al. 2014), not only to better understand their involvement in pathological conditions, but also to develop more focused and effective treatments (Baijnath et al. 2022; Scifo et al. 2017; Shariatgorji et al. 2014).

Matrix‐assisted laser desorption/ionization mass spectrometry imaging (MALDI‐MSI) is a powerful label‐free analytical tool that allows mapping of the spatial distributions and the abundance of a wide variety of biomolecules in tissue samples (Caprioli et al. 1997). The technique can detect hundreds of molecules simultaneously in a single experiment, with subsequent in situ localization and relative quantitation in a tissue section (Norris and Caprioli 2013).

Sample preparation is one of the main challenges of studying signaling molecules in the brain by MALDI‐MSI. Immediately after euthanization, rapid molecular degradation may occur (Dienel 2020, 2021; Nylander et al. 1997; Schmidt et al. 1972; Skold et al. 2007). Snap‐freezing of tissues is a widely employed method used to reduce postmortem enzymatic activities. However, dissection and freezing of the brain may take time, and some enzymes may also be reactivated during thawing (Schmidt et al. 1972). To circumvent these drawbacks, several strategies have been adopted to stabilize tissue samples and limit the degradation of biomolecules. Among in vivo fixation techniques, focused microwave irradiation (FMW) to the brain, by which the tissues are rapidly heated to 85°C–95°C, was developed to irreversibly deactivate enzymes, preventing postmortem degradation of neurotransmitters and neuropeptides (Balcom et al. 1975; Blank et al. 1979; Nylander et al. 1997; O'Callaghan and Sriram 2004; Stenfors et al. 1995). Also, in situ freezing minimizes postmortem changes in the contents of cerebral labile neurotransmitters and metabolites (Hattori et al. 2010; Sugiura et al. 2012), as well as the use of enzyme inhibitors like 3‐mercaptopropionic acid, which is a glutamic acid decarboxylase inhibitor and prevents the postmortem increase in γ‐aminobutyric acid (GABA) levels (Belforte et al. 2010; Cai et al. 2014; van der Heyden and Korf 1978). However, these stabilization techniques may have limitations, such as toxicity, ethical restrictions, time consumption, and high costs (Svensson et al. 2009). Furthermore, overheating with subsequent destruction of the sample area or uneven heating throughout the tissue during FMW may result in reversible enzymatic activity (Che et al. 2005; Verhage et al. 2000).

In our previous works, we proposed an alternative tissue stabilizing technique, which uses a combination of heat and pressure under vacuum for the fixation of brain samples prior to peptidomic and proteomic profiling (Skold et al. 2007; Svensson et al. 2009). Heat stabilization is performed by rapid and efficient heat transfer through a lower and upper conductive heat block during vacuum application, ensuring homogenous heating at 95°C in any part of the sample. This approach reduces the degradation processes of proteins and peptides, as well as preserves their post‐translational modifications (Segerstrom et al. 2016; Skold et al. 2007; Svensson et al. 2009).

This work investigated in detail the postmortem degradation differences among heat‐stabilized (HS) and fresh frozen (FF) brain tissues for key neurotransmitters, metabolites, and neuropeptides using MALDI‐MSI analysis. Furthermore, the application of FF and HS tissue sample preparation protocols on the level of neurotransmitters and metabolites changes was studied on rat brain tissues affected by acute anesthesia, which is often administered before euthanization in animal experiments and might impact the signaling system in the brain. The MALDI‐MSI analysis showed that the heat treatment preserved the spatial information and effectively limited postmortem metabolic degradation of neurotransmitters and neuropeptides, resulting in informative molecular profiling, which can be used for understanding molecular changes in both healthy and disease conditions, as well as their modulation following drug treatment.

## 2 Experimental Section

### 2.1 Chemicals

All chemicals were purchased from Sigma‐Aldrich (Stockholm, Sweden) and were used without further purification unless otherwise stated. Isotope‐labeled neurotransmitters (dopamine‐1,1,2,2‐*d*_4_ • HCl (DA‐*d*_4_), 4‐hydroxy‐3‐methoxyphenyl‐*d*_*3*_‐acetic‐*d*_*2*_ acid (HVA‐*d*_5_), and γ‐aminobutyric acid‐2,2,3,3,4,4‐*d*_6_ (GABA‐*d*_6_)) were purchased from CDN Isotopes (Quebec, Canada). The reactive MALDI matrix 4‐(anthracen‐9‐yl)‐2‐fluoro‐1‐methylpyridin‐1‐ium iodide salt (FMP‐10) was purchased from Tag‐ON AB (Uppsala, Sweden). Isoflurane (Isoflo Vet) and medetomidine (Domitor Vet) were purchased from Orion Pharma Animal Health (Sollentuna, Sweden; RRID:SCR_003911).

### 2.2 Safety and Precautions

The chloroform ACS reagent (≥ 99.8%) and isoflurane were handled according to all safety precautions indicated by the manufacturer.

### 2.3 Animal Experiments

All animal experiments were approved by the Uppsala Animal Ethical Committee (permit number 5.8.18–00833/2017) and followed the guidelines of the Swedish Legislation on Animal Experimentation (Animal Welfare Act SFS 2018:1192) and the European Union Directive on the Protection of Animals Used for Scientific Purposes (Directive 2010/63/EU).

Female Long Evans rats (RjOrl:LE, Janvier Labs, Le Genest‐st‐Isle, France, RRID:RGD_151356971; *n* = 8 ovariectomized and *n* = 12 intact) at 11 weeks of age were used. After arrival, the animals were left undisturbed for 2 weeks to acclimatize to the facility and the reversed light/dark cycle (Arts et al. 2014). They were kept 3–4 per cage in transparent cages of type IV (59 × 38 × 20 cm) with raised lids containing wood chip bedding. For enrichment purposes, each cage had paper sheets (40 × 60 cm, Cellstoff, Papyrus) and a wood tunnel. The cages were kept on a reversed light/dark cycle (lights off at 6:00 am) with a masking background noise at a constant temperature (22°C ± 1°C) and humidity (50% ± 10%). The animals had access to rat chow (Type R36, Lantmännen, Kimstad, Sweden) and tap water ad libitum. No sample size calculation was performed. Instead, the number of animals was on the basis of experience from previous experiments (Vallianatou et al. 2024; Vallianatou et al. 2019; Vallianatou et al. 2021; Vallianatou et al. 2018). No exclusion criteria were predetermined, and no animal was excluded or died during experiments. No randomization was performed to allocate subjects in the study.

To test the suitability of the tissue sample preparation protocol for analyzing neurotransmitters and neuropeptides, eight ovariectomized rats were sacrificed by awake decapitation. Following decapitation, the brains were removed. Half of the brains (*n* = 4) were immediately frozen by immersion in −20°C dry ice–cooled isopentane for 1 min, while the other half (*n* = 4) were heat‐stabilized (Stabilizor T1 instrument, Denator AB, Sweden). For both treatments, the post‐mortem delay between decapitation and the initiation of freezing or heating was 1 min. All samples were then stored at −80°C until further use.

After the initial experiment, HS and FF fixation protocols were evaluated on brain tissues subjected to acute anesthesia. Twelve intact female rats were divided into four groups (*n* = 3/group). Six rats were sacrificed by awake decapitation, and six rats were subjected to isoflurane and medetomidine anesthesia prior to decapitation on the basis of a procedure used in a previous study (Tjernstrom et al. 2022). Anesthesia was induced in a box with 5% isoflurane (1:4 oxygen/air mixture). Once the animals were sufficiently anesthetized, they were injected with 0.05 mg/kg medetomidine and then placed back into the box where the isoflurane level was lowered to 1%. The animals were decapitated 20 min after the medetomidine injection. Following decapitation, the brains were removed within 1 min. Prior to storage at −80°C, the brains in each group (either those subjected to live decapitation or those anesthetized before decapitation) were divided. One half was immediately frozen by immersion in −20°C dry ice‐cooled isopentane for 1 min, while the other half was heat‐stabilized.

### 2.4 Heat Stabilization

Stabilization by conductive heat transfer was performed at 95°C with minimal compression and cavity vacuum, using the Stabilizor T1 instrument (Denator AB, Sweden). This process inactivates enzymes in the sample by a quick and strictly controlled increment of the temperature, resulting in rapid and uniform protein denaturation. The whole brains were stabilized in the device's “fresh structure preserve” mode. Samples were placed in a Maintainor Tissue card (Denator AB, Sweden) to ensure sufficient contact with the heat source, and the air was evacuated with a needle to reduce the risk of oxidation and ensure efficient heat transfer.

### 2.5 Cryosectioning

The rat brain samples were mounted in a cryostat microtome (Leica CM1900, Leica Microsystems, Wetzlar, Germany), attached by using drops of water, and sectioned at a thickness of 12 μm at ‐20°C upon 1‐hour conditioning. Tissue sections were thaw‐mounted onto pre‐cooled indium tin oxide (ITO) coated glass slides (Bruker Daltonics, Bremen, Germany) and stored at −80°C until further use. MALDI‐MSI for both neurotransmitters and neuropeptides was performed on coronal rat sections at four levels (distance from bregma): 2.28, −2.04, −2.92, and −5.64 mm (Paxinos and Watson 2014) (Figure S1) for the initial experiment comparing HS and FF. Anesthesia‐treated and non‐treated brains, subjected to HS or FF, were sectioned at 2.28 and −2.92 mm from bregma to visualize brain regions known to be affected by anesthesia (Irifune et al. 1997; Whittington and Virag 2006).

### 2.6 Chemical Derivatization of Dopaminergic, Serotonergic, and GABAergic Neurotransmitters and Their Metabolites

Neurotransmitters and their metabolites of dopaminergic and serotonergic metabolic pathways and GABA were visualized by a selective in situ chemical derivatization method recently developed (Shariatgorji et al. 2019; Shariatgorji et al. 2021). Briefly, sections were desiccated at room temperature for 20 min. In the optimized protocol, a mixture of deuterated internal standards (IS), consisting of 0.67 μg/mL DA‐*d*_*4*_, 0.33 μg/mL HVA‐*d*_*5*_, and 100 μg/mL GABA‐*d*_*6*_ in 50% methanol, was homogeneously sprayed over the tissues using an automated sprayer (TM‐Sprayer, HTX Technologies, Chapel Hill, NC, USA) under the following conditions to deposit 0.13, 0.06, and 19.1 ng/mm^2^ of IS, respectively, on samples: nozzle temperature was set at 90°C, with a nitrogen gas pressure of 6 psi, solvent flow rate of 70 μL/min, nozzle velocity of 1100 mm/min, six passes with horizontal/vertical rotation, and 2 mm track spacing. After the IS solution spraying, the reactive matrix FMP‐10 was dissolved in 70% acetonitrile (ACN) (4.4 mM, 5.5 mL) and sprayed over the tissue sections using the automated pneumatic sprayer (TM‐Sprayer, HTX Technologies) to coat the samples with 1.32 μg/mm^2^ of the reactive matrix FMP‐10 with the following parameters: nozzle temperature of 80°C, nitrogen gas pressure of 6 psi, solvent flow rate of 80 μL/min, nozzle velocity of 1100 mm/min, 20 horizontal passes with offsets, and 2 mm track spacing.

### 2.7 Sample Preparation for MALDI‐MSI Analysis of Neuropeptides

We used a slightly modified tissue‐washing protocol that was essential for reducing lipid‐related signal suppression and improving the signal‐to‐noise ratio for neuropeptides. As previously evaluated (Hulme et al. 2020), the chloroform wash proved to be the most effective method for the detection of endogenous neuropeptides. This approach significantly reduced peaks in the lipid mass range (*m/z* 700–1000), increased the abundance of signals at higher m/z values, and did not cause visible delocalization of neuropeptides. Furthermore, it allowed for the detection of the potassium adduct ion of phosphatidylcholine (PC) (34:1)+K^+^ (see below), used as a lock mass in the MS method across the entire tissue. Briefly, the slides were dried with a stream of nitrogen followed by vacuum desiccation for 20 min. The washing procedure was performed by fully immersing the glass slide with the tissue sections in a glass Petri dish containing 45 mL of chloroform for 35 s, with gentle swirling and continual movement of the slide in the chloroform throughout the wash. The chloroform was changed between washing each slide. After the washing, the slide was immediately placed in a vacuum desiccator and dried for 15 min before MALDI matrix 2,5‐dihydroxybenzoic acid (DHB) at a concentration of 25 mg/mL in 50% ACN/0.2% trifluoroacetic acid (v/v) application. The DHB matrix solution was sprayed over the tissues using the automated sprayer (TM‐Sprayer, HTX Technologies), with the following conditions: the nozzle temperature was set at 85°C, with a nitrogen gas pressure of 6 psi, a flow rate of 120 μL/min, a nozzle velocity of 1200 mm/min with four passes, and 2 mm track spacing.

### 2.8 MALDI‐MSI Analysis and Data Processing

Prior to the MALDI‐MSI analysis, optical images of the tissues were acquired using a standard flatbed scanner (Seiko Epson, Japan). All MALDI‐MSI experiments were performed on a solariX 7 T‐2ω MALDI‐Fourier‐transform ion cyclotron resonance (FTICR) mass spectrometer (Bruker Daltonics, Bremen, Germany) equipped with a Smartbeam II 2 kHz laser. The analyses were performed in positive ion mode, at a lateral resolution of 150 μm, with 100 laser shots per position. The laser power was optimized at the start of each run and then held constant during the experiment. The data were acquired in the mass range of *m/z* 100–1500 using 2 million data points, and the mass range of *m/z* 450–3000 using 514 thousand data points for neurotransmitters and neuropeptides, respectively. The ion transfer optics parameters were set as follows: funnel RF amplitude 120.0 Vpp, RF amplitude TOF 350.0 Vpp, TOF 0.8 ms (1 ms for neuropeptides analyses), and RF frequency transfer optic 4 MHz. The Q1 mass was set to *m/z* 379 for analysis of neurotransmitters and *m/z* 650 for neuropeptides. The MS methods were calibrated externally using red phosphorus and internally using lock masses according to the analysis: the FMP‐10 cluster ion (*m/z* 555.2231) for analysis of neurotransmitters and the [PC (34:1)+K]^+^ molecular ion (*m/z* 798.54096) for analysis of neuropeptides (Guenther et al. 2011). Tissue sections were analyzed in a random order to prevent any possible bias due to matrix degradation or variation in mass spectrometer sensitivity.

MSI data were visualized using FlexImaging (v.4.1, Bruker Daltonics) and the SCiLS software package (v.2020a Pro, Bruker Daltonics). Neurotransmitters and metabolites data were normalized to the appropriate deuterated standard. Neuropeptides data were normalized to the root‐mean‐square (RMS) of all data points. For each compound, the mean ion intensity within the region of interest (either the whole tissue section or a specific brain region) was used for data exploration (Tables S2, S3 and S5–S7) and further relative quantitation. Neurotransmitter turnover ratios were calculated using the average intensity ratio between the metabolite and the neurotransmitter for HS and FF brain tissues (Barton et al. 2008).

### 2.9 Liquid Chromatography–Tandem Mass Spectrometry Peptide Identification

Given the challenges associated with acquiring MALDI‐tandem MS (MS/MS) spectra of neuropeptides directly from tissue sections (Vu et al. 2021), the verification of specific neuropeptides involved comparison using a previously published rat brain tissue study from us (Hulme et al. 2020) subjected to homogenization and analysis via liquid chromatography (LC)‐MS/MS. Briefly, neuropeptides were extracted from brain tissue and identification was achieved using a nano‐LC system coupled with an electrospray quadrupole time‐of‐flight (Q‐TOF) mass spectrometer (Synapt G2‐Si, Waters Corp., Manchester, UK). The obtained raw data were transformed into Mascot Generic Format and subjected to X! Tandem search against the UniProt *Rattus norvegicus* database. Additionally, neuropeptides demonstrate distinct regional localization, which serves as supplementary confirmation of their identity. Therefore, our identification approach relies on the superior mass accuracy achieved via FTICR, corroborated by LC–MS/MS data, along with the precise regional distribution patterns exhibited by the respective neuropeptides.

### 2.10 Statistical Analysis

For MALDI‐MSI experiments comparing FF and HS tissues, the differences of mean values of the metabolites between the two sample preparation techniques were analyzed for statistical significance using the parametric two‐tailed Student's *t*‐test for independent groups (GraphPad Prism 7.05, GraphPad Software, San Diego, CA).

For anesthesia‐treated and non‐treated brains subjected to HS or FF, principal component analysis (PCA) for defined neurotransmitter data sets was performed using the SIMCA software (v.15.0, Sartorius Stedim Biotech GmbH, Göttingen, Germany). The Gaussian distribution of the data was tested using the Shapiro–Wilk normality test. Because of the small sample size within the HS and FF groups of anesthesia‐treated and non‐treated brain tissue samples (*n* = 3/group), the nonparametric Mann–Whitney *U*‐test without continuity correction was used for the statistical analysis. Results were expressed as mean ± standard deviation for parametric statistics and median with interquartile range for nonparametric statistics. Analysis with *p* ≤ 0.05 was considered statistically significant. No test for outliers was conducted.

## 3 Results

### 3.1 Effect of Heat Stabilization on Tissue Integrity

High temperature heating of the brain tissue caused some changes in the fine structure of the tissue. The HS tissues were slightly compressed (dorsal‐ventral) during the heat stabilization using the Stabilizer T1 instrument. Similar to previously reported (Goodwin et al. 2010), small voids were observed in the tissue (Figure S2) and the heat treatment also appeared to dry the tissue slightly.

Although we did not explicitly perform stability assays with chemical standards, previous studies employing rapid microwave‐induced and conductive heat transfer stabilization methods have confirmed the stability of neurotransmitters (dopamine, serotonin, norepinephrine, and acetylcholine) and neuropeptides (substance P, dynorphins, and enkephalins) under conditions analogous to our HS procedure. These results indicate that rapid heating effectively preserves the integrity of these molecules by preventing enzymatic degradation (Goodwin et al. 2010; Kanazawa and Jessell 1976; Nylander et al. 1997; Segerstrom et al. 2016; Wasek et al. 2018).

### 3.2 Normalization to Internal Standards Improved Pixel‐To‐Pixel Variation of Small Molecule Neurotransmitters

We evaluated the chemical derivatization method with FMP‐10 and confirmed that the derivatization of the target molecules occurred in the HS tissues (Table S1). Unlike the visualization of neurotransmitters in the FF tissue, RMS normalization of data originated from HS tissue did not effectively reduce ion suppression artifacts and appeared almost like the non‐normalized image (Figure S3). To improve the visualization quality, we optimized a mixture of IS suitable for the normalization of the ion intensity of the neurotransmitters detected in the HS tissue. The selection of the IS for the normalization of each endogenous neurotransmitter is crucial for optimized imaging (Kallback et al. 2012; Macha et al. 2024). The IS must be selected in the form with the same number of derivatized functional groups and the same type of structural rearrangements to ensure similar ionization/desorption properties.

In our study, the neurotransmitters of dopaminergic and serotonergic pathways and their downstream metabolism products were normalized against the deuterated IS DA‐*d*_4_, since they share very similar chemical structures as derivatized molecules, while HVA and GABA were normalized against the labeled compound HVA‐*d*_5_ and GABA‐*d*_6_, respectively. The IS normalization provided a more uniform signal intensity across the brain regions, both in FF and HS. Although for FF samples the IS normalized images appeared similar in effectiveness to RMS normalization, the IS normalization of HS tissues reduced the effects of the ion suppression arising from the HS tissues inhomogeneity and improved the level of pixel‐to‐pixel variation, which provided a higher definition of the brain structure with a uniform distribution of the signals (Figures S3 and S4).

### 3.3 Heat Stabilized‐Induced Effects on Neurotransmitter Metabolites

**FIGURE 1:**
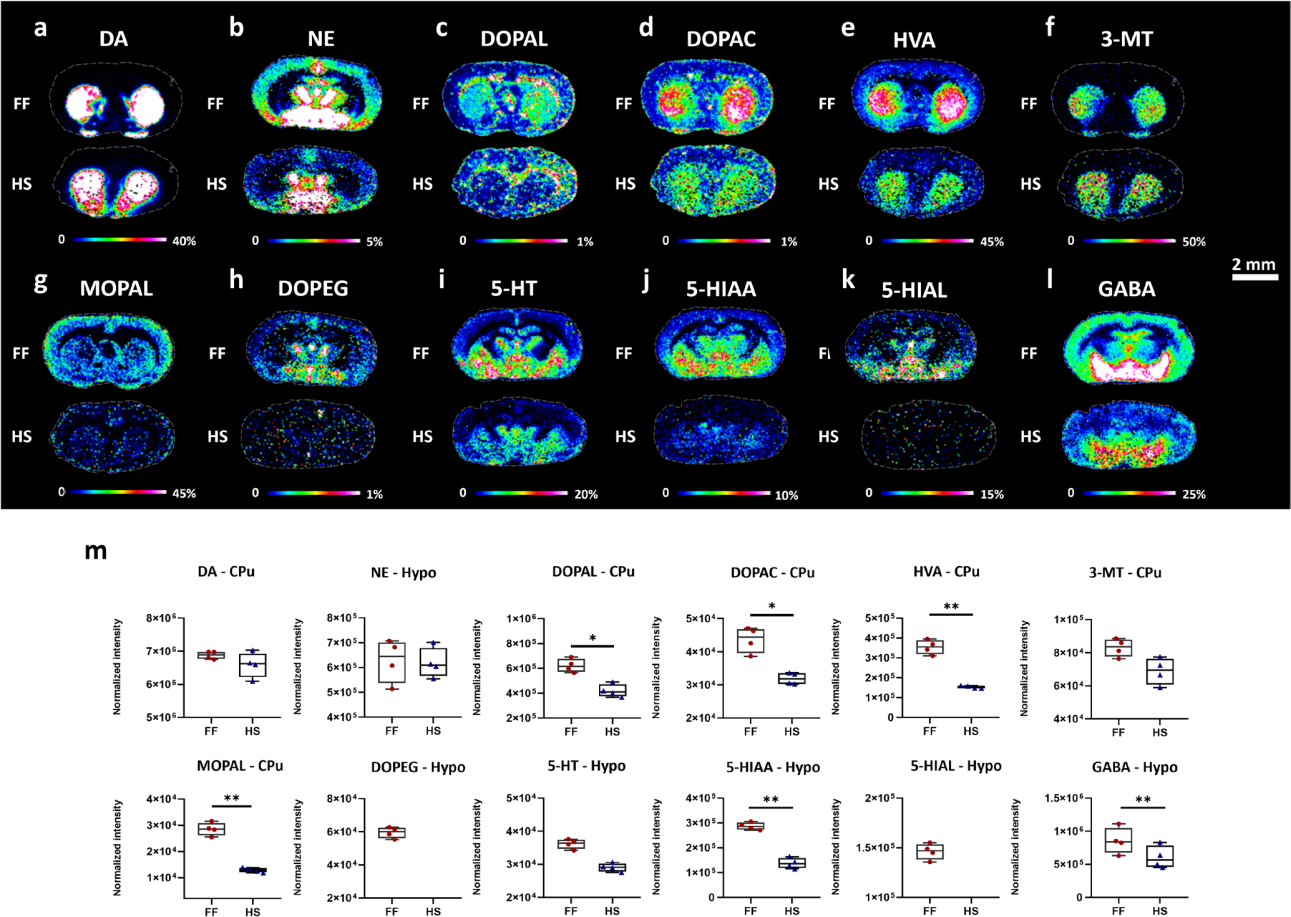
MALDI‐MS images of neurotransmitters and related metabolites acquired from FF and HS tissue sections. (a) DA, double derivatized, (b) NE, double derivatized, (c) DOPAL, double derivatized, (d) DOPAC, double derivatized, (e) HVA, single derivatized, (f) 3‐MT, single derivatized, (g) MOPAL, single derivatized, (h) DOPEG, double derivatized, (i) 5‐HT, single derivatized, (j) 5‐HIAA, single derivatized, (k) 5‐HIAL, single derivatized, (l) GABA, single derivatized. (a, c‐g), coronal rat brain section at bregma 2.28 mm, and (b, h–l), coronal rat brain section at bregma −2.04 mm. Scale bar, 2 mm. Color scale bars are shown as percentage of maximum intensity. (m) Box plots reflecting normalized average intensity of DA, DOPAL, DOPAC, HVA, 3‐MT, and MOPAL in caudate‐putamen (CPu) at bregma 2.28 mm, and NE, DOPEG, 5‐HT, 5‐HIAA, 5‐HIAL, and GABA in hypothalamus (Hypo) at bregma −2.04 mm of HS and FF brain sections (*n* = 4). Statistical comparison of DA, NE, 5‐HT, and their metabolites between HS and FF protocols is presented in Table 1. Lateral resolution, 150 μm. Data were normalized against IS, that is, DA‐*d*_*4*_ for DA, DOPAL, DOPAC, 3‐MT, MOPAL, 5‐HT, NE, DOPEG, 5‐HIAL, and 5‐HIAA, HVA‐*d*_*5*_ for HVA, and GABA‐*d*_*6*_ for GABA. The annotation of the brain regions is represented in Figure S5. 3‐MT, 3‐methoxytyramine; 5‐HIAA, 5‐hydroxyindoleacetic acid; 5‐HIAL, 5‐hydroxyindoleacetaldehyde; 5‐HT, serotonin; CPu, caudate‐putamen; Hypo, hypothalamus; DA, dopamine; DOPAC, 3,4‐dihydroxyphenylacetic acid; DOPAL, 3,4‐dihydroxyphenylacetaldehyde; DOPEG, 3,4‐dihydroxyphenylglycol; GABA, γ‐aminobutyric acid; HVA, homovanillic acid; MOPAL, 3‐methoxy‐4‐hydroxyphenylacetaldehyde; NE, norepinephrine. The *p*‐values were obtained using a parametric, two‐tailed Student's t‐test for independent groups (*n*=4). *p ≤ 0.05, **p ≤ 0.01.

MALDI‐MSI was applied to map the lateral distribution of bioactive compounds (Table S1), including DA and serotonin (5‐hydroxytryptamine, 5‐HT) metabolic systems and GABA (Figure 1) in both FF and HS brain tissue regions. Within the dopaminergic metabolic pathway, the detection of DA (Figure 1a) and norepinephrine (NE) (Figure 1b) was comparable between HS and FF. However, the different stabilization processes affected the related metabolites. We found that concentrations of the main metabolites of DA, 3,4‐dihydroxyphenylacetaldehyde (DOPAL) (Figure 1c), 3,4‐dihydroxyphenylacetic acid (DOPAC) (Figure 1d), and HVA (Figure 1e), were 1.5, 1.4, and 2.1‐fold higher, respectively, in the caudate‐putamen of FF compared to HS brain tissues (Table S2). The level of 3‐methoxytyramine (3‐MT) (Figure 1f) in both FF and HS tissues was similar, while the metabolite 3‐methoxy‐4‐hydroxyphenylacetaldehyde (MOPAL) (Figure 1g) was 2.3‐fold higher in the FF (Table S2) compared to HS brains. Moreover, we found that the metabolite of NE, dihydroxyphenylethylene glycol (DOPEG) (Figure 1h), was clearly detected in FF brains but not in HS tissues.

The effect of HS was further evaluated on the 5‐HT pathway. No significant differences in the detection of 5‐HT (Figure 1i) between HS and FF tissues were seen in the hypothalamus, thalamic nuclei, and caudate‐putamen brain regions. On the contrary, the metabolite 5‐hydroxyindoleacetic acid (5‐HIAA) (Figure 1j) was more than 2 times higher in FF brains (Table S2) in the hypothalamus, thalamic nuclei, and caudate‐putamen. The metabolite 5‐hydroxyindolealdehyde (5‐HIAL) (Figure 1k) was clearly detected in FF tissues but was not detected in HS sections. Overall, the results demonstrated increased abundance of DA and 5‐HT related metabolites in FF brain tissues that was also further supported by a reduced metabolite/neurotransmitter ratio in HS brain regions (Table S3). Lastly, we found that GABA (Figure 1l) levels were significantly lower (1.4‐fold, Table S2) in the hypothalamus and caudate‐putamen brain regions of HS tissues. Table 1 presents the statistical comparison of DA, NE, 5‐HT, and their metabolites between HS and FF protocols. *p*‐values, t‐values, and degrees of freedom were obtained using a parametric, two‐tailed Student's *t*‐test for independent groups. Figure S6 shows the MALDI‐MSI images of neurotransmitters for the four experimental replicates of the FF and HS samples.

**TABLE 1:** Statistical comparison of DA, NE, 5‐HT, and their metabolites between heat‐stabilized (HS) and fresh frozen (FF) protocols.

Bregma 2.28 mm						
Neurotransmitter	DA	DOPAL	DOPAC	MOPAL	3‐MT	HVA
Brain tissue	Caudate‐putamen (CPu)					
	Higher in FF compared to HS					
Degree of freedom (df)	4.001	4.450	3.545	3.564	4.067	3.240
*t* value	2.018	3.208	5.511	9.203	2.425	5.474
*p* value	0.1137	0.0281	0.0045	0.0013	0.7131	0.0098
*p* value symbol	ns	*	*	**	ns	**

Bregma −2.04 mm						
Neurotransmitter	NE	DOPEG	5‐HT	5‐HIAA	5‐HIAL	GABA
Brain tissue	Hypothalamus (Hypo)					
	Higher in FF compared to HS					
Degree of freedom (df)	2.169	ND in HS	4.001	3.745	ND in HS	3.311
*t* value	1.463		1644	6.080		10.310
*p* value	0.2717		0.1756	0.0046		0.0013
*p* value symbol	ns	—	ns	**	—	**

Brain tissue	Thalamic nuclei (TN)					
	Higher in FF compared to HS					
Degree of freedom (df)	4.001	ND in HS	3.570	2.916	ND in HS	4.001
*t* value	1.722		2.332	9.156		1.722
*p* value	0.1602		0.0880	0.0012		0.1602
*p* value symbol	ns	—	ns	**	—	ns

Brain tissue	Caudate‐putamen (CPu)					
	Higher in FF compared to HS					
Degree of freedom (df)	ND	ND	4.001	2.957	ND in HS	3.846
*t* value			1.722	9.184		6.366
*p* value			0.1623	0.0014		0.0039
*p* value symbol	—	—	ns	**	—	**

*Note:* *p*‐values, *t*‐values, and degrees of freedom (df) were obtained using a parametric, two‐tailed Student's *t*‐test for independent groups (*n* = 4).

Abbreviations: 3‐MT, 3‐methoxytyramine; 5‐HIAA, 5‐hydroxyindoleacetic acid; 5‐HIAL, 5‐hydroxyindoleacetaldehyde; 5‐HT, serotonin; DA, dopamine; DOPAC, 3,4‐dihydroxyphenylacetic acid; DOPAL, 3,4‐dihydroxyphenylacetaldehyde; DOPEG, 3,4‐dihydroxyphenylglycol; GABA, γ‐aminobutyric acid; HVA, homovanillic acid; MOPAL, 3‐methoxy‐4‐hydroxyphenylacetaldehyde; ND, not detected; NE, norepinephrine; ns: not significant.

* *p* ≤ 0.05.

** *p* ≤ 0.01.

### 3.4 HS Enhanced the Detection of Neuropeptides

The effect of the HS was investigated by mapping neuropeptides in brain tissue by MALDI‐MSI. A high number of peptides (in total 44) were mapped across both HS and FF brain tissues, including peptides derived from proenkephalin (PENK), prodynorphin (PDYN), nociceptin, the cerebellin 1 precursor, as well as tachykinins, neuropeptide‐EI, and neurotensin (Table S4). In FF brain tissue, we recently reported 27 neuropeptides (Hulme et al. 2020). In the present study, we identified an additional 17 neuropeptides. The identification of the neuropeptides was on the basis of the ultra‐high mass accuracy provided by MALDI‐FTICR‐MS and subsequent confirmation by LC–MS/MS analysis (Hulme et al. 2020).

### 3.5 PENK Neuropeptides

**FIGURE 2:**
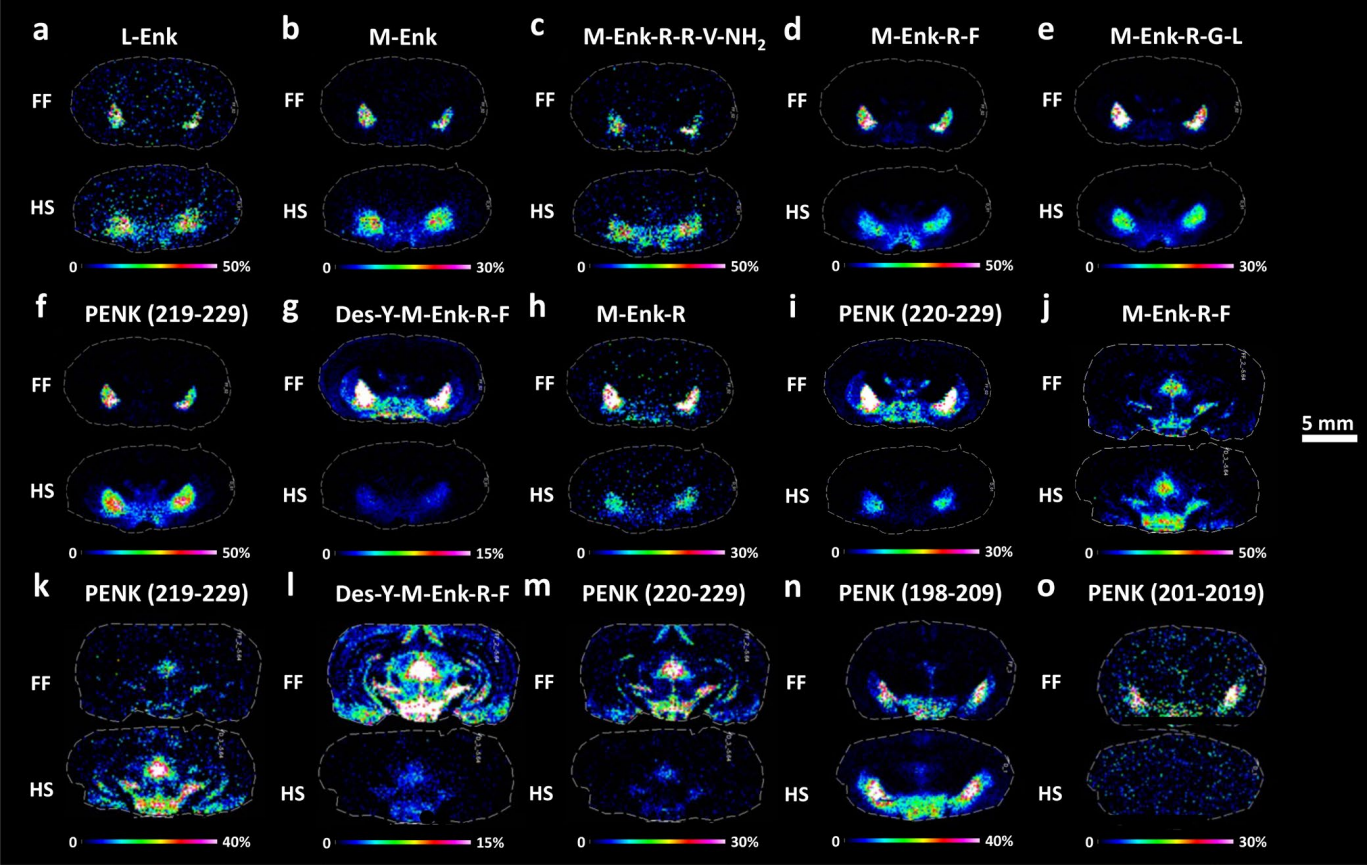
MALDI‐MS images of enkephalins in coronal rat brain sections. Imaging experiments compared the distribution of full‐length peptides (a–f, j, k, n) and related fragments (g–i, l, m, o) between FF and HS tissues (*n* = 4) at bregma −2.04 mm (a–i), −5.64 mm (j–m) and −2.92 mm (n, o). (a) Leu‐Enk, (b) Met‐Enk, (c) Met‐Enk‐Arg‐Arg‐Val‐NH_2_, (d, j) Met‐Enk‐Arg‐Phe, (e) Met‐Enk‐Arg‐Gly‐Leu, (f, k) PENK (219–229), (g, l) Des‐Y‐Met‐Enk‐Arg‐Phe, (h) Met‐Enk‐Arg, (i, m) PENK (220–229), (n) PENK (198–209), and (o) PENK (201–219). Scale bar, 5 mm. Color scale bars are shown as percentage of maximum intensity. Data were normalized against RMS. The statistical comparisons of PENK neuropeptides between HS and FF protocols are presented in Table 2. Lateral resolution, 150 μm. The annotation of the brain regions is represented in Figure S5. PENK, proenkephalin.

A group of endogenous opioid peptides derived from the PENK precursor were mapped across different brain regions from HS and FF coronal rat brain tissue sections. At the level −2.04 mm from bregma, Leu‐Enk (Figure 2a), Met‐Enk (Figure 2b), and Met‐Enk‐Arg‐Arg‐Val‐NH_2_ (metorphamide) (Figure 2c) were 2.8, 2.9, and 2.5‐fold higher, respectively, in the globus pallidus of HS tissues than FF (Table S5). Additionally, Met‐Enk‐Arg‐Phe (Figure 2d) and Met‐Enk‐Arg‐Gly‐Leu (Figure 2e) were abundantly present both in globus pallidus and caudate‐putamen of HS tissues. Instead, in FF tissues, the same PENK neuropeptides were clearly detected in globus pallidus but almost undetectable in the other brain regions. Moreover, PENK (219–229) (Figure 2f) was 3.9‐fold higher in globus pallidus of HS compared to FF (Table S5). The discrepancies may be due to the rapid enzymatic degradation of PENK neuropeptides and were only detected in the regions in which they were particularly abundant. In fact, the opposite was observed for the related shorter peptides, considered possible PENK degradation products, i.e., Des‐Tyr‐Met‐Enk‐Arg‐Phe, Met‐Enk‐Arg, and PENK (220–229) (Figure 2g–i) whose levels were 9.8, 1.9, and 7.9‐fold higher, respectively, in the globus pallidus of FF tissues than HS (Table S5). A similar trend was observed at the level −5.64 mm from bregma, at which the full‐length peptides Met‐Enk‐Arg‐Phe (Figure 2j) and PENK (219–229) (Figure 2k) were 2 and 6‐fold higher, respectively, in the periaqueductal gray and the substantia nigra of HS tissues (Table S5), while the peptide fragments Des‐Tyr‐Met‐Enk‐Arg‐Phe and PENK (220–229) were 7 and 4‐fold increased, respectively, in the same structures of FF tissues (Figure 2l,m; Table S5). Figure S7 shows the MALDI‐MSI images of PENK for the four experimental replicates of the FF and HS samples at bregma level −2.04 mm.

Furthermore, we observed that full length PENK (198–209) (Figure 2n) was 1.4‐fold higher in the amygdala of HS brains compared to FF tissues (Table S5). On the contrary, the degradation product PENK (201–209) (Figure 2o) was clearly detected in FF tissues and not detected in HS tissues coronal rat brain section at bregma −2.92 mm. Table 2 presents the statistical comparison of PENK neuropeptides between HS and FF protocols. *p*‐values, t‐values, and degrees of freedom were obtained using a parametric, two‐tailed Student's *t*‐test for independent groups.

**TABLE 2:** Statistical comparison of PENK neuropeptides between heat‐stabilized (HS) and fresh frozen (FF) protocols.

Bregma −2.04 mm									
Peptide	Leu‐Enk	Met‐Enk	Met‐Enk‐Arg‐Arg‐Val‐NH_2_	Met‐Enk‐Arg‐Phe	Met‐Enk‐Arg‐Gly‐Leu	PENK (219–229)	PENK Des‐Tyr‐ Met‐Enk‐Arg‐Phe	Met‐Enk‐Arg	PENK (220–229)
Brain tissue	Globus pallidus (GP)								
	Higher in HS compared to FF						Higher in FF compared to HS		
Degree of freedom (df)	3.623	4.282	4.681	6.001	3.001	4.759	5.034	3.011	6.001
*t* value	4.243	2.941	3.410	2.517	3.464	5.505	7.811	7.247	6.803
*p* value	0.0163	0.0389	0.0199	0.0455	0.0134	0.0032	0.0005	0.0054	0.0005
*p* value symbol	*	*	*	*	*	**	***	**	***

Brain tissue	Caudate‐putamen (CPu)								
	Higher in HS compared to FF						Higher in FF compared to HS		
Degree of freedom (df)	ND	ND in FF	ND in FF	ND in FF	ND in FF	ND in FF	3.007	ND	3.029
*t* value							6.001		8.506
*p* value							0.0092		0.0033
*p* value symbol							**	—	**

Brain tissue	Lateral hypothalamus (LH)								
	Higher in HS compared to FF						Higher in FF compared to HS		
Degree of freedom (df)	ND	ND	ND	5.020	1.142	—	3.684	ND	2.079
*t* value				0.698	0.564	—	5.940		3.264
*p* value				0.51662	0.3771	—	0.0052		0.0492
*p* value symbol				ns	ns	—	**	—	*

Bregma −5.64 mm				
Peptide	Met‐Enk‐Arg‐Phe	PENK (219–229)	PENK Des‐Tyr‐ Met‐Enk‐Arg‐Phe	PENK (220–229)
Brain tissue	Periaqueductal gray (PAG)			
	Higher in HS compared to FF		Higher in FF compared to HS	
Degree of freedom (df)	3.082	3.001	3.148	3.001
*t* value	3.938	17.701	11.880	3.566
*p* value	0.0278	0.0004	0.0010	0.0377
*p* value symbol	*	***	**	*

Brain tissue	Substantia nigra (SN)			
	Higher in HS compared to FF		Higher in FF compared to HS	
Degree of freedom (df)	3.001	4.371	3.105	3.148
*t* value	3.978	10.110	10.001	11.880
*p* value	0.284	0.0003	0.0018	0.0010
*p* value symbol	*	***	**	**

*Note:* *p*‐values, *t*‐values, and degrees of freedom (df) were obtained using a parametric, two‐tailed Student's *t*‐test for independent groups (*n* = 4).

Abbreviations: Enk, enkephalin; ND, not detected; ns, not significant; PENK, proenkephalin.

* *p* ≤ 0.05.

** *p* ≤ 0.01.

*** *p* ≤ 0.001.

There were no significant differences in the detection of PENK (114–133), PENK (212–229), and PENK (239–260) between FF and HS tissues (Figure S8).

### 3.6 PDYN, Tachykinin, and Nociceptin Neuropeptides

**FIGURE 3:**
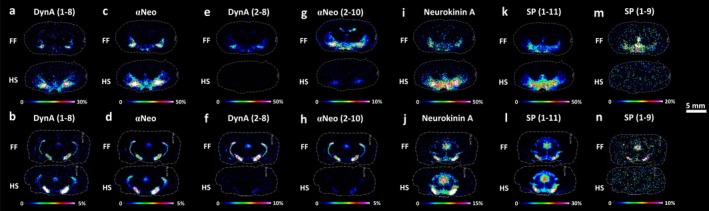
MALDI‐MS images of dynorphins and tachykinins in coronal rat brain sections. Imaging experiments compared the distribution of full‐length peptides (a–d, i–l) and related fragments (e–h, m, n) between FF and HS tissues (*n* = 4) at bregma −2.04 mm (a, c, e, g, i, k, m) and −5.64 mm (b, d, f, h, j, l, n) (*n* = 4). (a, b) DynA (1–8), (c, d) αNeo, (e, f) DynA (2–8), (g, h) αNeo (2–10), (i, j) Neurokinin A, (k, l) SP (1–11), and (m, n) SP (1–9). The statistical comparison of PDYN and tachykinin neuropeptides between HS and FF protocols is presented in Table 3. Scale bar, 5 mm. Color scale bars are shown as a percentage of maximum intensity. Data were normalized against RMS. Lateral resolution, 150 μm. The annotation of the brain regions is represented in Figure S5. Dyn, dynorphin; SP, substance P; αNeo, alpha‐neoendorphin.

The other group of opioid peptides derived from PDYN and the tachykinin neuropeptides was imaged at the selected brain levels and brain regions. Dynorphin A (DynA) (1–8) and α‐neoendorphin (α‐Neo) were 3.9‐fold and 2.3‐fold higher, respectively, in the lateral hypothalamus (−2.04 mm from bregma, Figure 3a,c; Table S6) and 3.7‐fold and 2.3‐fold higher, respectively, in the substantia nigra (−5.64 mm from bregma, Figure 3b,d; Table S6) in HS than in FF tissues. Otherwise, the peptide fragments, DynA (2–8) and α‐Neo (2–10) (Figure 3e–h), were approximately 10‐fold higher in the corresponding structures of the FF brains (Table S6). A similar trend was seen for dynorphin B (DynB) (1–13), DynB (1–6), DynB (15–28), and β‐neoendorphin (β‐Neo), showing higher levels in HS brains than FF tissues (1.66, 1.1, 2.01, and 5.4‐fold, respectively, Figure S8).

Regarding tachykinins, the levels of neurokinin A (Figure 3i,j) were on average 3‐fold higher across HS tissues (Table S6), and substance P (SP) (1–11) (Figure 3k,l) was higher in the lateral hypothalamus, the periaqueductal gray, and the substantia nigra of HS by 2.3‐, 2.1‐, and 1.8‐fold, respectively, at the level −2.04 and −5.64 mm from bregma (Table S6). On the contrary, SP (1–9) was detected only in FF tissues (Figure 3m,n). MALDI‐MSI visualization of PDYN and tachykinin in the four experimental replicates of FF and HS samples at bregma −2.04 mm is shown in Figure S9.

Notably, at the level −2.92 mm from bregma, the C‐terminal flanking peptide and neuropeptide‐γ were visualized for the first time and were exclusively detected in HS tissues (Figure S8). Additionally, nociceptin was also only visualized in HS tissues (Figure S8). Table 3 presents the statistical comparison of PDYN and tachykinin neuropeptides between HS and FF protocols. *p*‐values, t‐values, and degrees of freedom were obtained using a parametric, two‐tailed Student's *t*‐test for independent groups.

**TABLE 3:** Statistical comparison of PDYN and tachykinin neuropeptides between heat‐stabilized (HS) and fresh frozen (FF) protocols.

Bregma −2.04 mm							
Peptide	DynA (1–8)	α‐Neo	DynA (2–8)	αNeo (2–10)	Neurokinin A	SP (1–11)	SP (1–9)
Brain tissue	Lateral hypothalamus (LH)						
	Higher in HS compared to FF		Higher in FF compared to HS		Higher in HS compared to FF		Higher in FF compared to HS
Degree of freedom (df)	3.663	4.379	3.011	6.001	5.644	3.890	ND in HS
*t* value	8.054	6.956	7.584	8.156	11.410	2.490	
*p* value	0.0019	0.0016	0.0052	0.0002	0.0001	0.0692	
*p* value symbol	**	**	**	***	***	ns	

Bregma −5.64 mm							
Brain tissue	Periaqueductal gray (PAG)						
	Higher in HS compared to FF		Higher in FF compared to HS		Higher in HS compared to FF		Higher in FF compared to HS
Degree of freedom (df)	2.930	3.001	5.432	3.007	2.868	4.019	ND in HS
*t* value	8.893	3.566	3.771	4.025	6.315	4.747	
*p* value	0.0033	0.0377	0.0112	0.0274	0.0092	0.0089	
*p* value symbol	**	*	*	*	**	**	

Brain tissue	Substantia nigra (SN)						
	Higher in HS compared to FF		Higher in FF compared to HS		Higher in HS compared to FF		Higher in FF compared to HS
Degree of freedom (df)	3.660	3.988	4.013	4.015	4.654	3.856	ND in HS
*t* value	3.743	2.841	6.535	4.701	6.308	2.962	
*p* value	0.0235	0.0470	0.0018	0.0092	0.0019	0.0434	
*p* value symbol	*	*	**	**	**	*	

*Note:* *p*‐values, *t*‐values, and degrees of freedom (df) were obtained using a parametric, two‐tailed Student's *t*‐test for independent groups (*n* = 4).

Abbreviations: ND, not detected; ns, not significant.

* *p* ≤ 0.05.

** *p* ≤ 0.01.

*** *p* ≤ 0.001.

### 3.7 Other Neuropeptides

**FIGURE 4:**
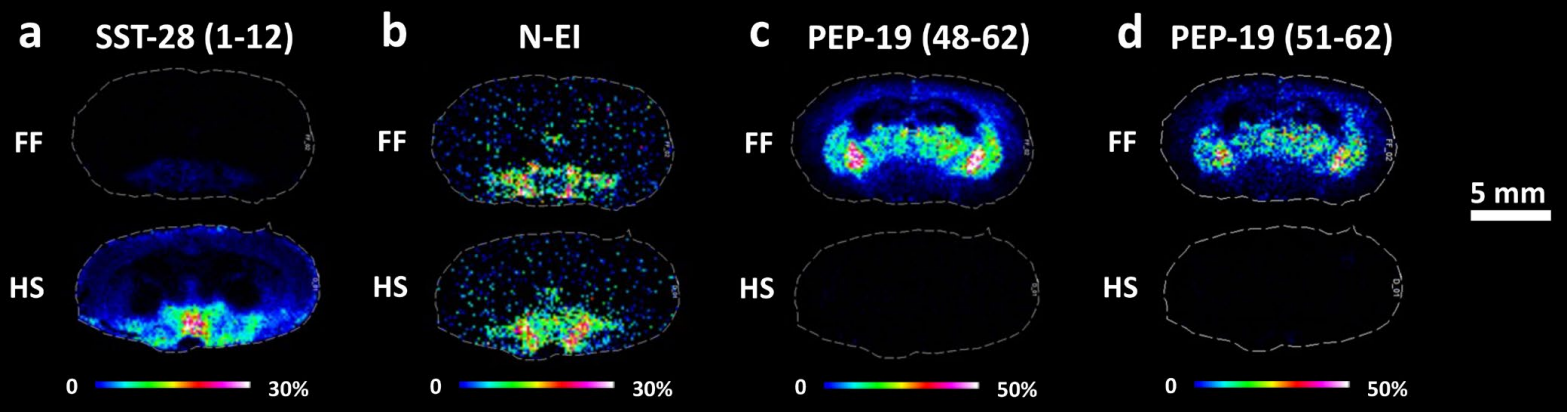
Molecular distributions determined by MALDI‐MSI in coronal rat brain sections comparing FF with HS tissues. (a) SST‐28 (1–12), (b) N‐EI, (c) PEP‐19 (48–62), and (d) PEP‐19 (51–62). Sections obtained at bregma −2.04 mm. Scale bar, 5 mm. Color scale bars are shown as a percentage of maximum intensity. Data were normalized against RMS. Lateral resolution, 150 μm. The statistical comparisons of somatostatin 28 (1–12) and neuropeptide EI between HS and FF protocols are presented in Table 4 (*n* = 4). The annotation of the brain regions is represented in Figure S5. N‐EI, neuropeptide EI; PEP‐19, purkinje cell protein 4; SST, somatostatin.

MALDI‐MSI revealed the presence of other neuropeptides derived from several precursors possessing different neuromodulatory functions. Somatostatin 28 (SST‐28) (1–12) is the bifunctional hormone released by the monobasic and dibasic cleavages of the precursor prosomatostatin. Neuropeptide EI (N‐EI) is derived from the melanin concentrating hormone (MCH) precursor. In this investigation, the biologically active peptides SST‐28 (1–12) (Figure 4a) and N‐EI (Figure 4b) were 14 and 1.5‐fold increased, respectively, in the hypothalamus of HS tissues compared to the FF brains (Table S7). Table 4 presents the statistical comparison of Somatostatin 28 (1–12) and Neuropeptide EI between HS and FF protocols. *p*‐values, t‐values, and degrees of freedom were obtained using a parametric, two‐tailed Student's *t*‐test for independent groups. Further evidence of the prevention of postmortem degradation by HS was demonstrated by exclusive detection of PEP‐19 (48–62) (Figure 4c) and PEP‐19 (51–62) (Figure 4d) i.e., fragments of the full‐length PEP‐19 (6.7 kDa neuronal calmodulin‐binding polypeptide) (Skold et al. 2006) in FF tissue. Figure S10 shows the MALDI‐MSI maps of Somatostatin 28 and PEP‐19 for the four experimental replicates of the FF and HS samples at bregma −2.04 mm. Prohormone convertase 1 (PC1) (619–628) was not detected in HS tissues, while there were no statistically significant differences in the detection of cerebellin neuropeptide in thalamic nuclei between the two methods of stabilization (Figure S11).

**TABLE 4:** Statistical comparison of Somatostatin 28 (1–12) and Neuropeptide EI between heat‐stabilized and fresh frozen protocols.

Bregma −2.04 mm		
Peptide	SST‐28 (1–12)	N‐EI
Brain tissue	Hypothalamus (Hypo)	
	Higher in HS compared to FF	
Degree of Freedom (df)	3.128	3.985
*t* value	14.801	0.0282
*p* value	0.0005	0.0784
*p* value symbol	***	ns

*Note:* *p*‐values, *t*‐values, and degrees of freedom (df) were obtained using a parametric, two‐tailed Student's *t*‐test for independent groups (*n* = 4).

Abbreviations: ns, not significant; SST, somatostatin.

*** *p* ≤ 0.001.

### 3.8 Effect of Anesthesia on Neurotransmitter Levels

**FIGURE 5:**
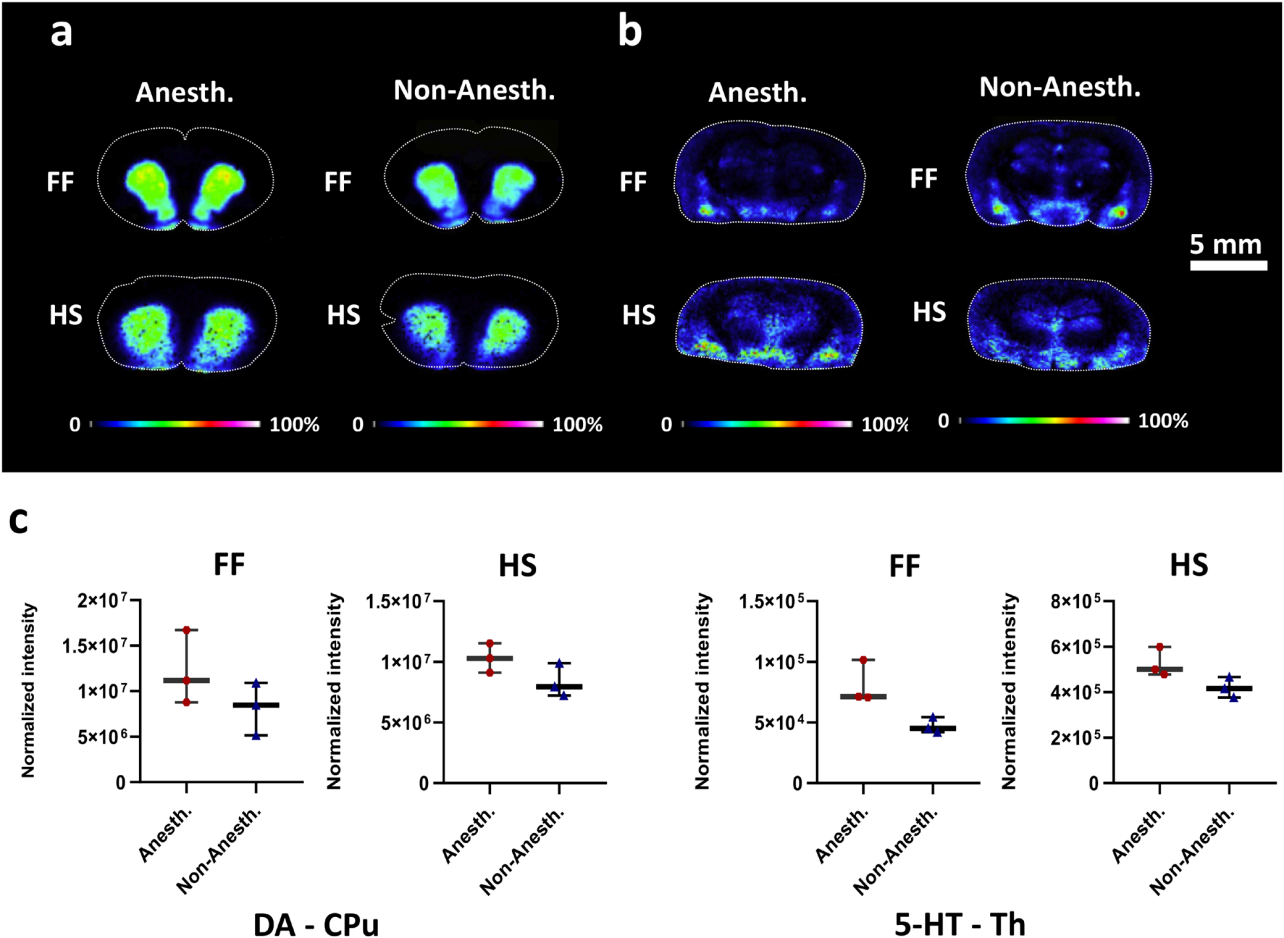
Imaging experiments comparing the effect of anesthesia on neurotransmitter levels in FF and HS coronal rat brain sections. (a) DA (at bregma 2.28 mm) and (b) 5‐HT (at bregma −2.92 mm) comparing the effect of anesthesia in FF with HS tissues. Color scale bars are shown as a percentage of maximum intensity. Lateral resolution, 150 μm. Data were normalized against the IS DA‐*d*_4_. (c) Box plots reflecting normalized average intensity of DA in caudate‐putamen (at bregma 2.28 mm) and 5‐HT in thalamus (at bregma −2.92 mm) comparing the effect of anesthesia in FF and HS brain tissues. All results are expressed as median with interquartile range. Statistical analysis was performed by nonparametric Mann–Whitney *U*‐test, without continuity correction. In the CPu, the effect of anesthesia on DA levels was not significant (*p* = 0.2, Mann–Whitney U = 1) in both FF and HS groups. Similarly, in the thalamus, the effect of anesthesia on 5‐HT levels was not significant (*p* = 0.1, Mann–Whitney U = 0) in both FF and HS groups. FF (fresh frozen)‐anesthetized (*n* = 3), non‐anesthetized (*n* = 3); HS (heat‐stabilized)‐anesthetized (*n* = 3), non‐anesthetized (*n* = 3). 5‐HT, serotonin; Anesth., anesthetized; CPu, caudate‐putamen; DA, dopamine; FF, fresh frozen; HS, heat stabilized; Non‐Anesth., non‐anesthetized; Th, thalamus.

Brain tissue samples from the non‐anesthetized animals and the animals subjected to acute anesthesia were prepared with the developed HS and conventional FF protocols for analysis of neurotransmitters. The effect of anesthesia on dopaminergic and serotonergic systems was evaluated in six selected brain regions, including the caudate‐putamen, septum, hippocampus, thalamus, hypothalamus, and amygdala that might be affected by anesthesia. Multivariate data analysis differentiated groups of HS and FF brain tissue samples with displaying reduced variations between HS compared to FF samples (Figure S12). Individual subgroups comprising anesthetized and non‐anesthetized brains showed the subdivision, particularly in the caudate‐putamen, septum, and thalamus (Figure S12). Subsequent statistical analysis using the nonparametric Mann–Whitney *U*‐test displayed in the aforementioned regions modified levels of neurotransmitters without statistical significance but with an increasing trend in the abundance of DA and 5‐HT (Figure 5, Figure S13) and their metabolites, including DOPAL, DOPAC, HVA, NE, and 5‐HIAA, mainly in anesthetized animals (Figure S13).

## 4 Discussion

In the present study, we evaluated the effectiveness of rapid and uniform heating of brain tissue to inactivate postmortem enzymatic activity, thereby preventing the degradation of the neuronal chemical messengers, and we compared the method to conventional snap freezing. The HS tissue sample preparation protocol allowed for the visualization of neurotransmitters and neuropeptide distribution across the different brain regions and minimized postmortem changes that better reflected the in vivo brain metabolic state of both neurotransmitters and neuropeptides. The HS procedure did not degrade neurotransmitters, nor cause detectable delocalization of the molecules, and the tissue integrity alteration did not affect the MSI results at our experimental lateral resolution.

To ensure the highest quality data visualization, the data normalization to the appropriate internal standards represents a proper strategy. This approach is beneficial for absolute quantitation experiments (Kallback et al. 2020) and for reducing ion suppression effects caused by different chemical compositions and structural environments in a brain tissue section (Kallback et al. 2016; Kallback et al. 2012). For efficient normalization, several parameters were taken into account when selecting the internal standards, that is, (i) the structural similarity between the labeled and unlabeled compounds, (ii) the numbers of derivatized functional groups (phenolic and/or primary amines) of both internal standards and endogenous compounds, and (iii) the types of structural rearrangements (loss of the hydrogen ion or/and the methyl group) that occur in multi‐derivatized molecules, resulting in single charged ions in MALDI (Shariatgorji et al. 2019; Shariatgorji et al. 2021). In this way, we achieved an optimized IS normalization for each neurotransmitter that exhibited a superior reduction in ion suppression artifacts compared to RMS normalization in HS brain tissues.

Several previous studies have found that euthanizing animals with FMW irradiation improved regional measurements of brain labile neurotransmitters (Bertrand et al. 1994; Schmidt et al. 1972). In this regard, both FMW fixation and HS have been proposed in combination with MALDI‐MSI to assess highly labile small molecules in tissues (Barre et al. 2018; Sugiura et al. 2014; Sugiura et al. 2016). Our results extend to the previous studies and present an alternative HS sample preparation approach for MALDI‐MSI to improve the visualization of labile neurotransmitters in brain tissues.

In contrast to recent investigations using FMW for measuring biogenic monoamines (Groppetti et al. 1977; Wasek et al. 2018), the HS method did not significantly increase the DA and NE levels. The treatment, however, had a profound effect on the related metabolites (DOPAL, DOPAC, HVA, MOPAL, and DOPEG) that were all significantly lower in HS tissues, showing a reduced turnover ratio in the HS brains. Most notable was the effect of heat stabilization of tissue on the turnover of 5‐HT. We observed a significant change between HS and FF tissues of the 5‐HT metabolites 5‐HIAA and 5‐HIAL. These results may indicate a reduction in the 5‐HT degradation following HS. This is in contrast to previous findings using brain fixation by the FWM technique, which assumed a steady state level of 5‐HT (Stenfors et al. 1995; Wasek et al. 2018). Thus, combining the HS method with MALDI‐MSI prevented metabolic artifacts and conducted more accurate biogenic monoamine determination in brain tissues. Furthermore, the sustained levels of neurotransmitters combined with significantly reduced downstream metabolite levels under HS conditions align with previous findings demonstrating rapid enzymatic inactivation and consequent reduced neurotransmitter metabolism following FMW (Wasek et al. 2018). Thus, the observed metabolite reductions are likely due to reduced metabolic turnover rather than decreased metabolite stability.

The effect of HS was also pronounced for GABA levels, which were increased postmortem in FF brains, most likely because of maintained postmortem activity of glutamate decarboxylase. Hence, the current study confirmed that the glutamate decarboxylase enzyme was effectively inhibited by HS, as reported in brain regions thermally stabilized by FMW (Balcom et al. 1975; Wasek et al. 2018).

Investigating the distribution and the abundance of neuropeptides in brain tissues is essential to highlight alterations in neurological diseases. It has been shown that following tissue sampling, active enzymes continue their proteolytic processes to generate peptide fragments that do not reflect the in vivo state of neuropeptide composition (Skold et al. 2002; Skold et al. 2007; Svensson et al. 2009). Thus, rapid and controlled heating of intact tissue is desirable to prevent brain peptidome degradation (Svensson et al. 2009; Svensson et al. 2007).

The RMS approach effectively normalized the spectra and reduced the ion suppression caused by the physical and structural properties of HS samples. Moreover, following the heat treatment, no delocalization or diffusion of the neuropeptides occurred, and the neuropeptide distributions observed in our experiments were consistent with the distribution previously reported using both MALDI‐MSI and other techniques (Fallon and Leslie 1986; Hanrieder et al. 2012; Hulme et al. 2020; Kallback et al. 2012; Shults et al. 1984; Taban et al. 2007).

Using the HS method, postmortem proteolytic activity of enzymes degrading PENK and PDYN neuropeptides was significantly inhibited, shown by a reduction of peptide degradation fragments. The change in PENK peptide degradation levels varied between the brain structures. For example, the distribution of Met‐Enk‐Arg‐Phe, Met‐Enk‐Arg‐Gly‐Leu, and PENK (219–229) was abundant in the globus pallidus of both HS and FF tissues; however, they were detected only in the caudate‐putamen of the HS tissues. Moreover, we detected the low‐abundance neuropeptides C‐terminal flanking peptide, neuropeptide‐γ, and nociceptin, exclusively in HS tissues, highlighting their susceptibility to enzymatic degradation.

The increased neuropeptide signals observed in HS‐treated tissue are consistent with previous MALDI‐MSI studies demonstrating that heat stabilization rapidly and uniformly inactivates proteolytic enzymes, significantly reducing neuropeptide degradation (Goodwin et al. 2010; Sturm et al. 2013; Svensson et al. 2009). Although subtle structural changes remain possible, these prior reports suggest minimal impact on analyte accessibility. Furthermore, our previous LC–MS/MS analyses support the interpretation that enhanced neuropeptide signals are mainly due to better preservation rather than altered accessibility (Adachi et al. 2005; Skold et al. 2007; Svensson et al. 2007; Svensson et al. 2003).

The proposed protocols were also used to study potential neurotransmitter changes due to the acute anesthesia in several brain regions. The results showed less variance in the HS data compared to those of FF brain tissues, supporting the effect of heat stabilization in terms of reduced postmortem metabolism of neurotransmitters and degradation of biomolecules (Skold et al. 2007; Svensson et al. 2009). However, no significant effects on neurotransmitter levels were observed.

In conclusion, a MALDI‐MSI approach was developed to highlight the effectiveness of rapid and uniform heating of brain tissues to inactivate native enzymatic activity and prevent the postmortem degradation of the neuronal chemical messengers. MALDI‐MSI mapped the spatial distribution of small molecule neurotransmitters and neuropeptides in heat‐treated rat brain sections. Despite changes to the tissue morphology and ion suppression artifacts, the spatial information of the molecules was not altered. The HS brain sample preparation protocol's suitability was also demonstrated in the experimental animal study of anesthesia administration, where no significant effects on neurotransmitters were revealed, but HS reduced variations between samples. These results enforce the potential of the heat treatment for sample preparations in MALDI‐MSI to provide a realistic snapshot of the levels of key signaling molecules in the brain, enhancing the information about their roles in both healthy and diseased conditions, as well as their modulation following pharmacological treatment.

## Author Contributions

**Emanuela Salviati:** conceptualization, investigation, formal analysis, methodology, validation, writing – original draft, writing – review and editing. **Dominika Luptáková:** conceptualization, methodology, investigation, validation, formal analysis, writing – original draft, writing – review and editing. **Anna Nilsson:** methodology, validation, writing – review and editing. **Reza Shariatgorji:** methodology, validation, writing – review and editing. **Pietro Campiglia:** conceptualization, resources, writing – review and editing. **Nikita Tjernström:** methodology, validation, writing – review and editing. **Erika Roman:** conceptualization, methodology, resources, validation, writing – review and editing, visualization, project administration, supervision, funding acquisition. **Per E. Andrén:** conceptualization, methodology, funding acquisition, validation, visualization, writing – review and editing, project administration, supervision, resources.

## Conflicts of Interest

A.N., R.S., and P.E.A. are co‐founders and shareholders in Tag‐ON AB.

## Supporting information


Data S1.

## Data Availability

The data that support the findings of this study are available from the corresponding author upon reasonable request.
