# Supplementary material for: Preserving Neuronal Chemical Messengers: Heat Stabilization Versus Snap Freezing for Improved MALDI Mass Spectrometry Imaging of Brain Tissues
Source: J Neurochem. 2025 Jun 16;169(6):e70122. doi: 10.1111/jnc.70122 (PMC12169076; doi:10.1111/jnc.70122)
Supplement: Supplementary file 1 — Data S1. [file JNC-169-0-s001.pdf]

Supplementary Material

Preserving neuronal chemical messengers: Heat stabilization vs. snap freezing for improved MALDI mass spectrometry imaging of brain tissues

Emanuela Salviati[1,2], Dominika Luptáková[1,3], Anna Nilsson[1], Reza Shariatgorji[1], Pietro Campiglia[2], Nikita Tjernström[4], Erika Roman[4,5], Per E. Andrén[1]*

1. Department of Pharmaceutical Biosciences, Spatial Mass Spectrometry, Science for Life Laboratory, Uppsala University, BMC 591, SE-75124, Uppsala, Sweden
2. Department of Pharmacy, University of Salerno, I-84084, Fisciano, (SA), Italy
3. Institute of Microbiology of the Czech Academy of Sciences, Vídeňská 1083, Prague 142 00, Czech Republic
4. Department of Pharmaceutical Biosciences, Neuropharmacology and Addiction, Uppsala University, BMC 591, SE-75124, Uppsala, Sweden
5. Department of Anatomy, Physiology and Biochemistry, Swedish University of Agricultural Sciences, Box 7011, SE-75007 Uppsala

* **Corresponding Author.** Phone: 46-70-167 9334. E-mail: per.andren@uu.se

Content

Supplementary Figures S1 – S13 Page 2 – 15
Supplementary Tables S1 – S7 Page 16 – 25
Reference Page 25

Supplementary Figures

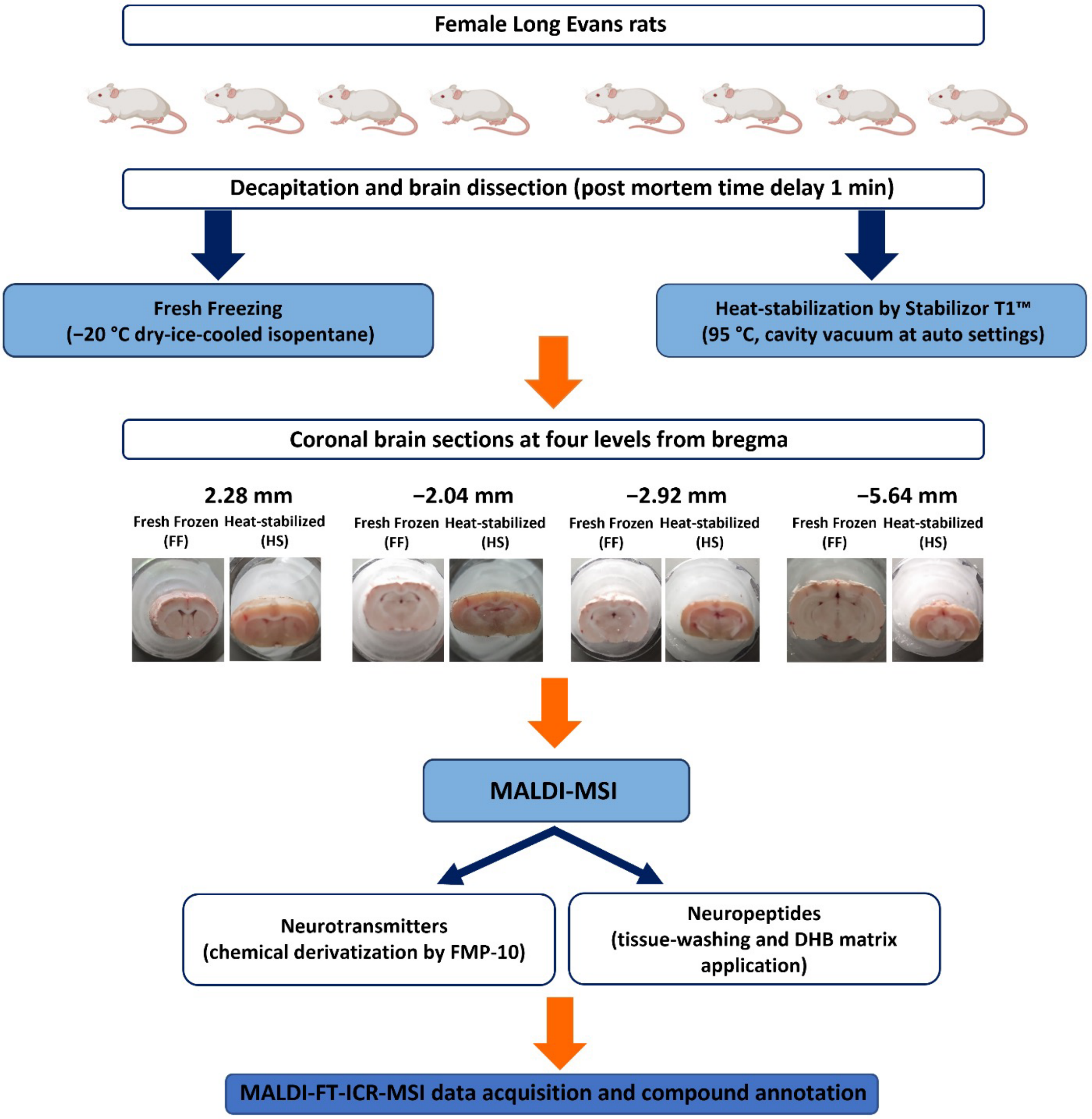

Figure S1. Schematic of the experiments performed in rat brains to monitor preservation of neurotransmitters and neuropeptides from postmortem degradation, comparing fresh freezing and heat stabilization as tissue fixation approaches.

Eight rats were sacrificed by decapitation and the brains were quickly removed. Four brains were immediately frozen by immersion in -20°C dry-ice-cooled isopentane and stored at -80 °C, the other four brains were heated at 95 °C within 1 min following decapitation with minimal compression and cavity vacuum, using the Stabilizor T1 instrument. MALDI-MSI for both neurotransmitters and neuropeptides were performed on coronal rat sections at four levels, distance from bregma: 2.28 mm, -2.04 mm, -2.92 mm, -5.64 mm (Paxinos & Watson 2014). Abbreviations: FF, fresh frozen; HS, heat-stabilized.

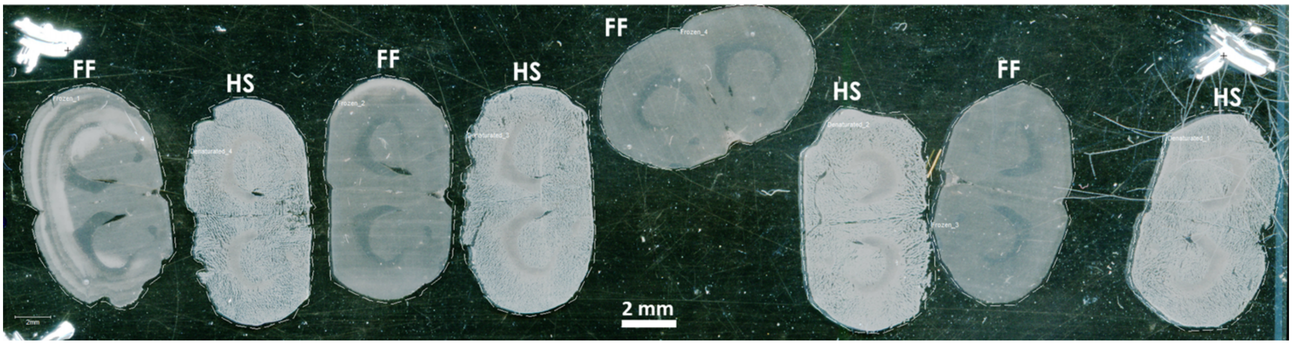

Figure S2. Optical images of coronal rat brain tissue sections from four heat treated brains and four fresh frozen brains.

The heat stabilized tissue sections appear dryer, their edges are slightly jagged and some voids can be observed (distance from bregma, 2.28 mm) (Paxinos & Watson 2014). Abbreviations: FF, fresh frozen; HS, heat-stabilized.

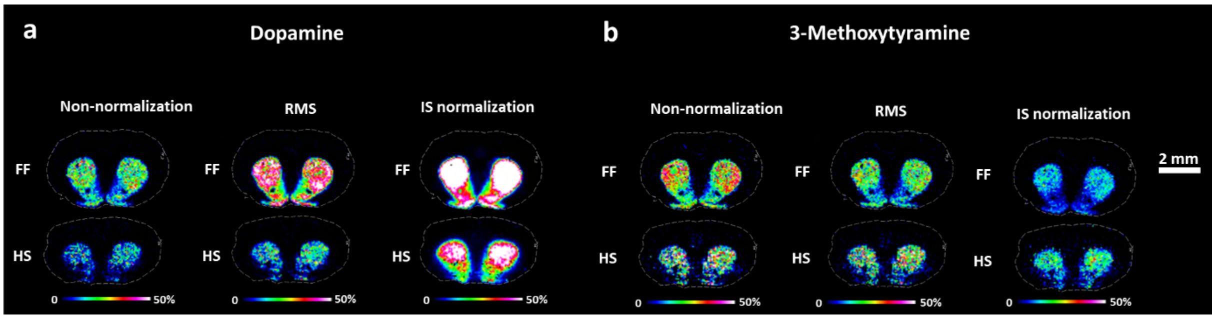

Figure S3. Investigation of different normalization methods of data originated from fresh frozen and heat stabilized tissues.

MALDI-MS images of double derivatized dopamine (**a**) and single derivatized 3-methoxytyramine (**b**) in coronal rat brain sections comparing non-normalized, RMS, and DA-d_4 normalized MSI data in FF and HS tissues (at bregma 2.28 mm). MALDI-MSI data were acquired at 150 µm lateral resolution. Color scale bars are shown as percentage of maximum intensity. Scale bar, 2 mm. Abbreviations: IS, internal standard (DA-d_4, dopamine-1,1,2,2-d_4 • HCl); RMS, root-mean square normalization; FF, fresh frozen; HS, heat-stabilized.

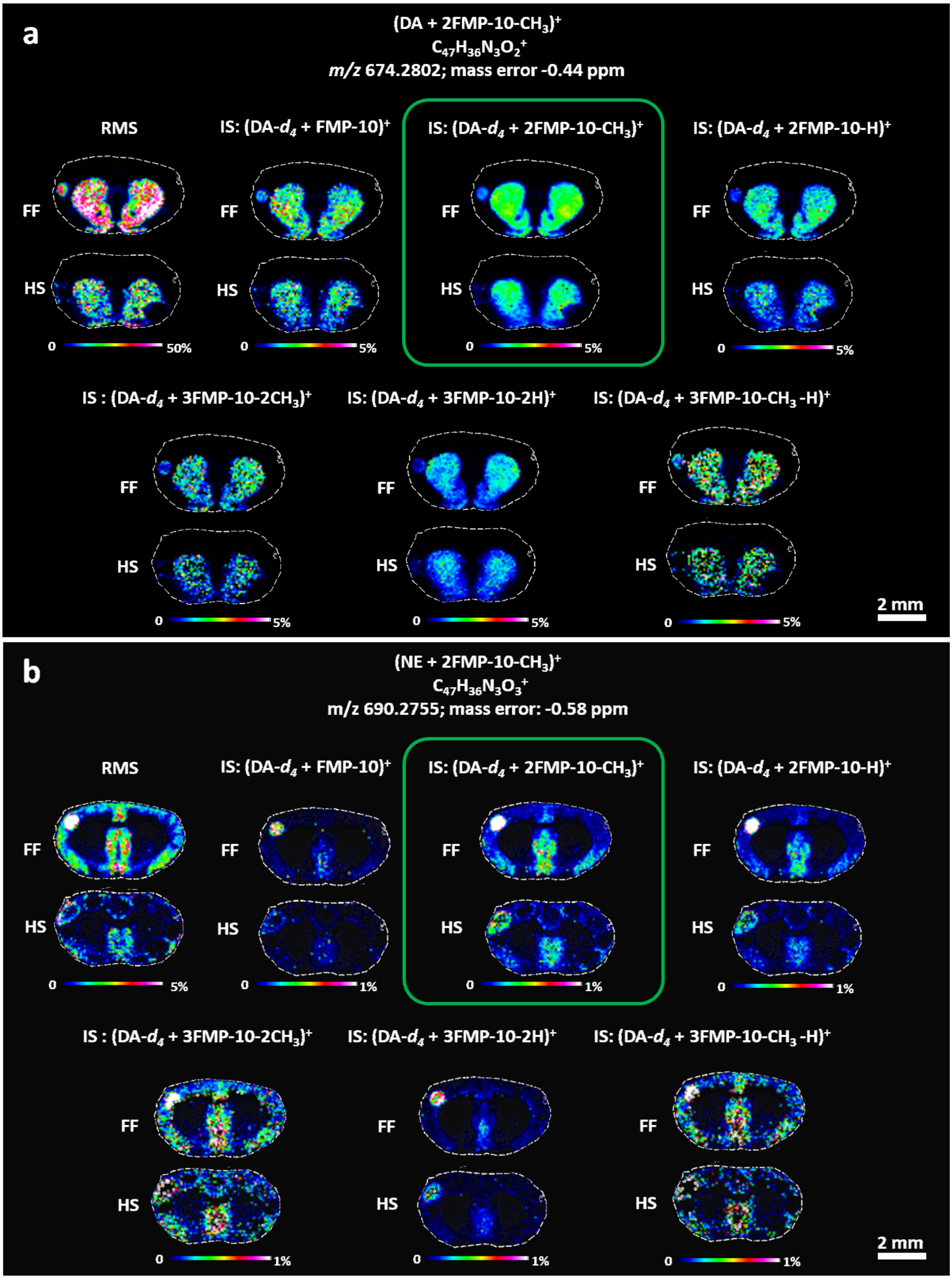
a
(DA + 2FMP-10-CH3)+
C47H36N3O2+
m/z 674.2802; mass error -0.44 ppm
RMS
IS: (DA-d4 + FMP-10)+
IS: (DA-d4 + 2FMP-10-CH3)+
IS: (DA-d4 + 2FMP-10-H)+
FF
HS
0
50%
5%
IS : (DA-d4 + 3FMP-10-2CH3)+
IS: (DA-d4 + 3FMP-10-2H)+
IS: (DA-d4 + 3FMP-10-CH3 -H)+
2 mm
b
(NE + 2FMP-10-CH3)+
C47H36N3O3+
m/z 690.2755; mass error: -0.58 ppm
1%

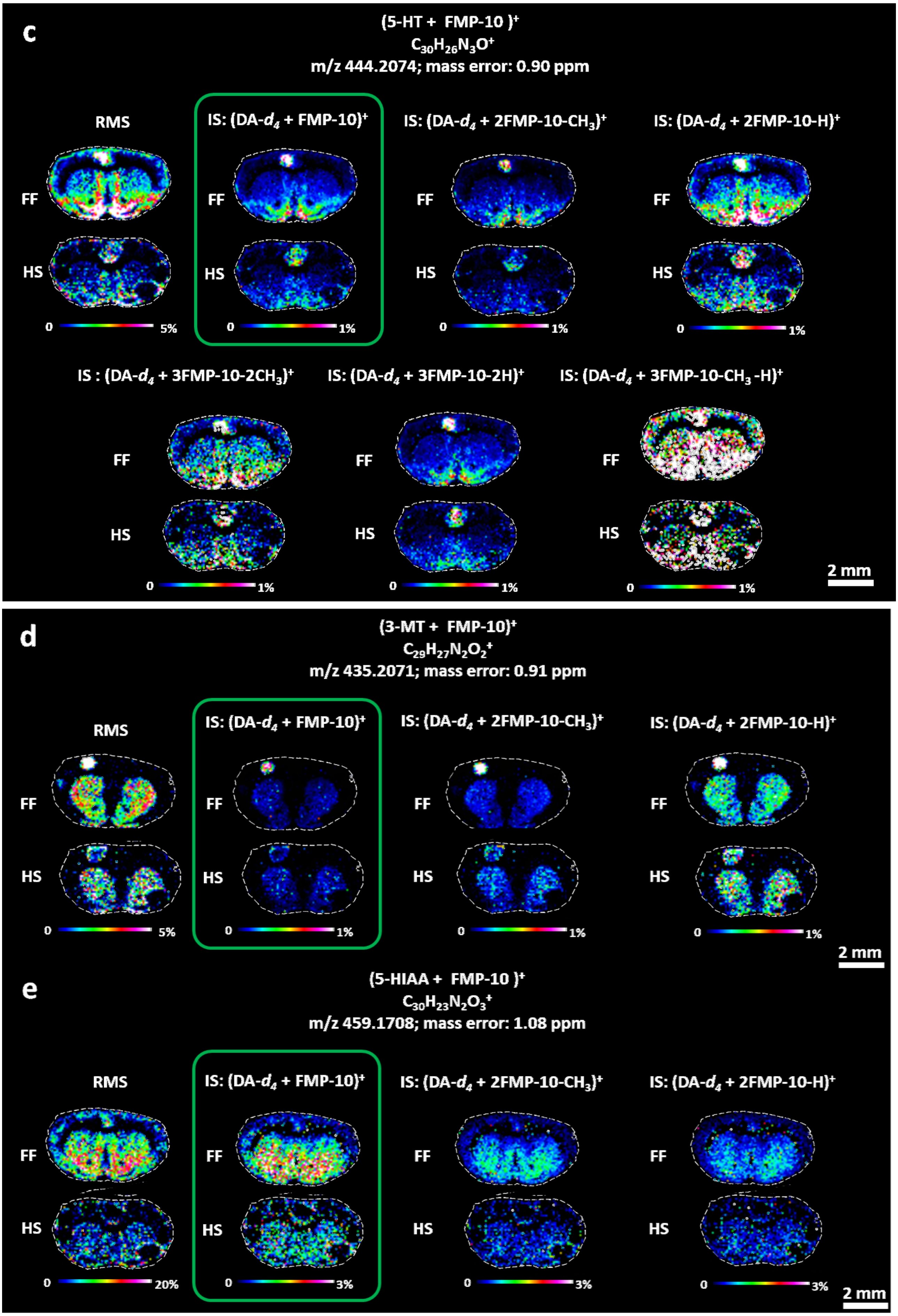
c
(5-HT + FMP-10)+
C30H26N3O+
m/z 444.2074; mass error: 0.90 ppm
RMS
IS: (DA-d4 + FMP-10)+
IS: (DA-d4 + 2FMP-10-CH3)+
IS: (DA-d4 + 2FMP-10-H)+
FF
HS
0
5%
1%
IS : (DA-d4 + 3FMP-10-2CH3)+
IS: (DA-d4 + 3FMP-10-2H)+
IS: (DA-d4 + 3FMP-10-CH3 -H)+
2 mm
d
(3-MT + FMP-10)+
C29H27N2O2+
m/z 435.2071; mass error: 0.91 ppm
RMS
IS: (DA-d4 + FMP-10)+
IS: (DA-d4 + 2FMP-10-CH3)+
IS: (DA-d4 + 2FMP-10-H)+
FF
HS
0
5%
1%
2 mm
e
(5-HIAA + FMP-10)+
C30H23N2O3+
m/z 459.1708; mass error: 1.08 ppm
RMS
IS: (DA-d4 + FMP-10)+
IS: (DA-d4 + 2FMP-10-CH3)+
IS: (DA-d4 + 2FMP-10-H)+
FF
HS
0
20%
3%
2 mm

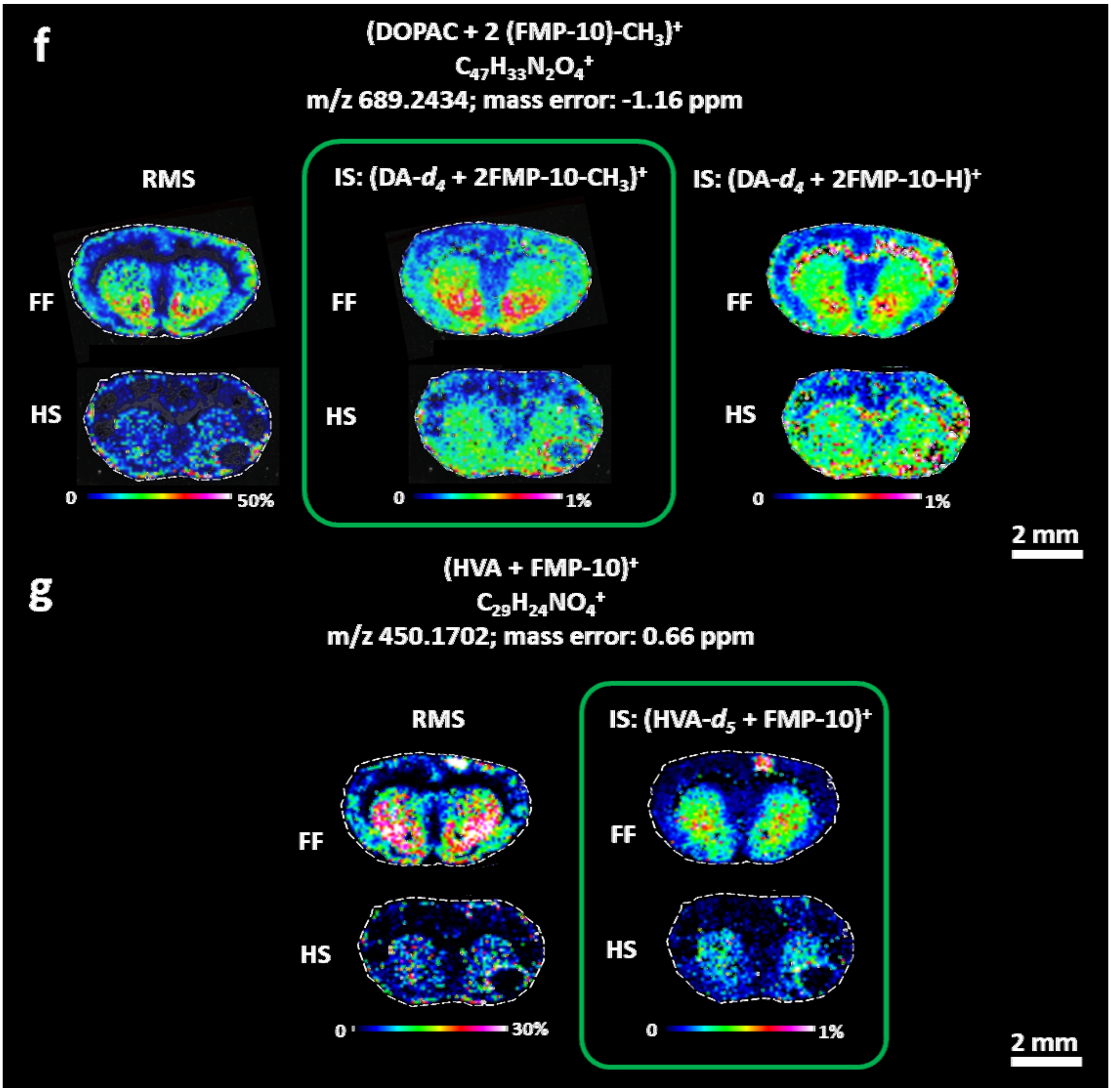
f
(DOPAC + 2 (FMP-10)-CH3)+
C47H33N2O4+
m/z 689.2434; mass error: -1.16 ppm
RMS
IS: (DA-d4 + 2FMP-10-CH3)+
IS: (DA-d4 + 2FMP-10-H)+
FF
HS
0
50%
1%
2 mm
g
(HVA + FMP-10)+
C29H24NO4+
m/z 450.1702; mass error: 0.66 ppm
RMS
IS: (HVA-d5 + FMP-10)+
30%
1%
2 mm

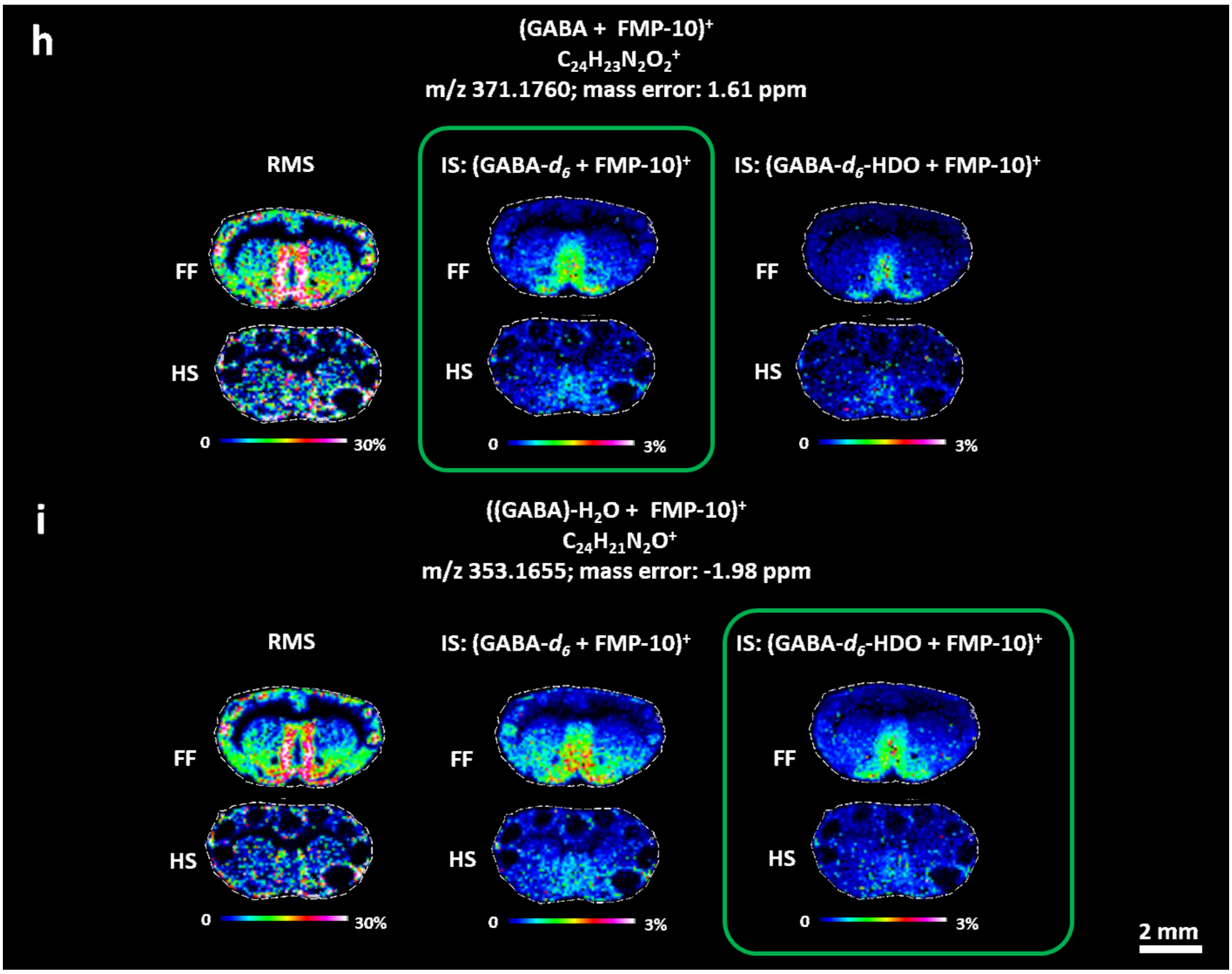

Figure S4. MALDI-MSI of fresh frozen and heat-stabilized coronal rat brain sections comparing root mean square (RMS) with internal standard (IS) normalization of neurotransmitter data.

Tissue distribution of (**a**) double derivatized DA normalized by RMS or by different forms of derivatized DA-d_4, (**b**) double derivatized NE normalized by RMS or by different forms of derivatized DA-d_4, (**c**) single derivatized 5-HT normalized by RMS or by different forms of derivatized DA-d_4, (**d**) single derivatized 3-MT normalized by RMS or by different forms of derivatized DA-d_4, (**e**) single derivatized 5-HIAA normalized by RMS or by different forms of derivatized DA-d_4, (**f**) double derivatized DOPAC normalized by RMS or by different forms of derivatized DA-d_4, (**g**) single derivatized HVA normalized by RMS or by different forms of derivatized HVA-d_5, (**h**) single derivatized GABA normalized by RMS or by different forms of derivatized GABA-d_6, and (**i**) single derivatized GABA-H_2O normalized by RMS or by different forms of derivatized GABA-d_6. Abbreviations: IS, internal standard normalization; RMS, root-mean square normalization; FF, fresh frozen; HS, heat-stabilized; DA, Dopamine; NE, Norepinephrine; 5-HT, Serotonin; 3-MT, 3-methoxytyramine; 5-HIAA, 5-hydroxyindoleacetic; DOPAC, dihydroxyphenylacetic acid; HVA, homovanillic acid; GABA, γ-aminobutyric acid; DA-d_4, dopamine-d_4; HVA-d_5, Homovanillic acid-d_5. (FMP-10), single derivatized; (2FMP-10-CH_3), double derivatized with the loss of methyl group; (2FMP-10-H), double derivatized with the loss of hydrogen ion. (3FMP-10-2CH_3), triple derivatized with the loss of two methyl groups; (3FMP-10-2H), triple derivatized with the loss of two proton; (3FMP-10-CH_3-H), triple derivatized with the loss one methyl group and one hydrogen ion. Scale bar, 2 mm. Colour scale bars are shown as percentage of maximum intensity. Lateral resolution, 150 µm; distance from bregma 2.28 mm.

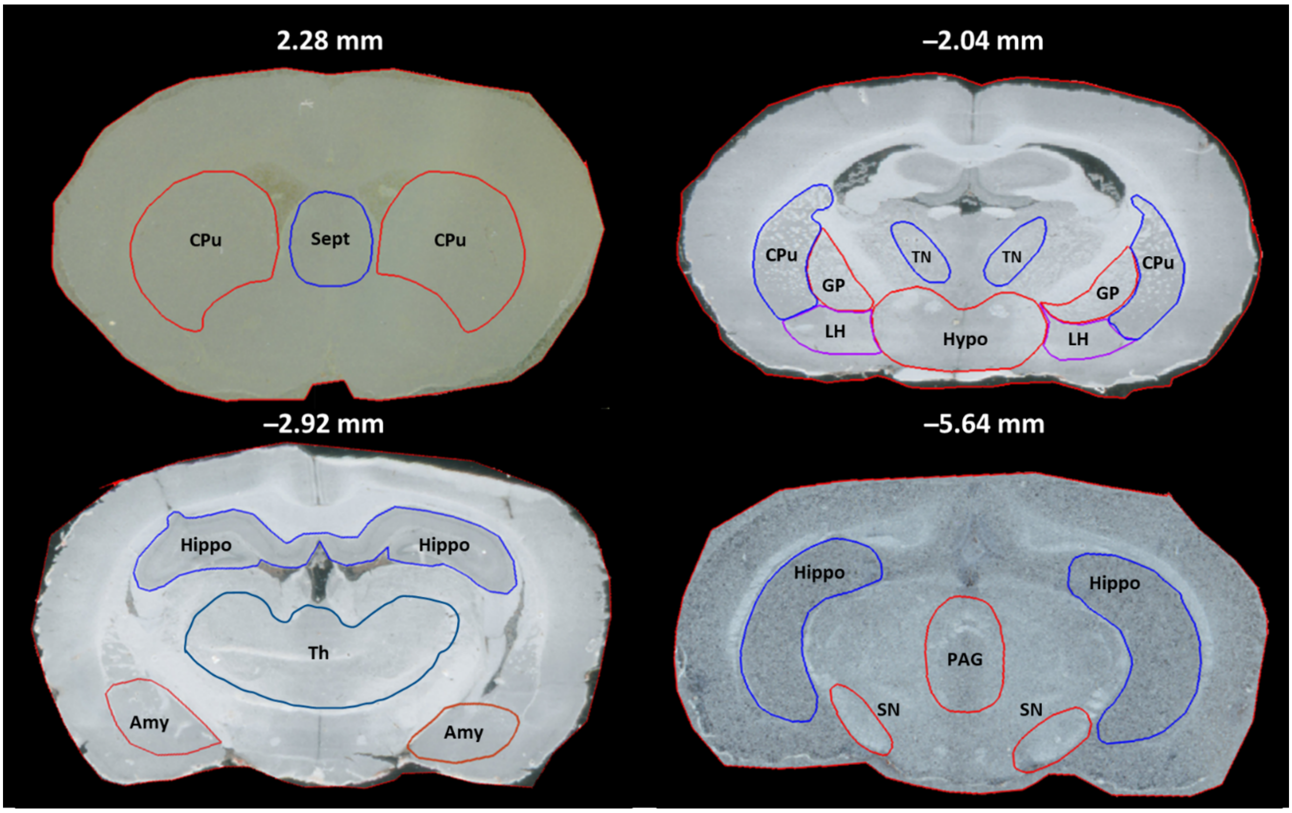

Figure S5. Annotation of the brain regions at bregma 2.28 mm, -2.04 mm, -2.92 mm, and -5.64 mm.

Abbreviations. CPu, Caudate-putamen; Sept, Septum; GP, Globus pallidus; TN, Thalamic nuclei; Th, Thalamus; Hypo, Hypothalamus; LH, Lateral hypothalamus; Amy, Amygdala; Hippo, Hippocampus; SN, Substantia nigra; PAG, Periaqueductal gray. Scale bar, 2 mm.

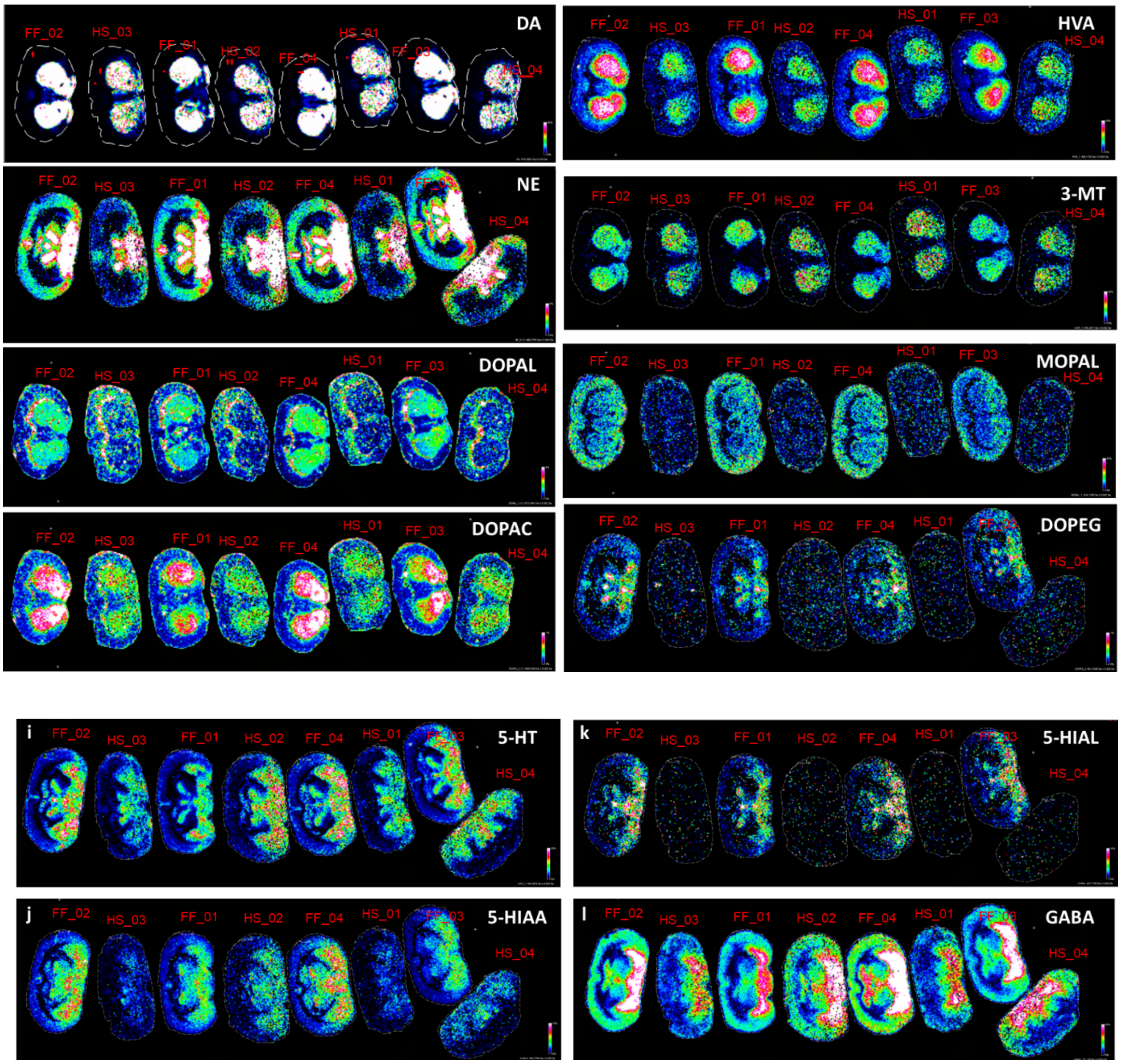

Figure S6. MALDI-MSI images of neurotransmitters for the four experimental replicates of the FF and HS samples.

(**a**) DA, double derivatized, (**b**) NE, double derivatized, (**c**) DOPAL, double derivatized, (**d**) DOPAC, double derivatized, (**e**) HVA, single derivatized, (**f**) 3-MT, single derivatized, (**g**) MOPAL, single derivatized (**a, c-g**, coronal rat brain section at bregma 2.28 mm). (**h**) DOPEG, double derivatized, (**i**) 5-HT, single derivatized, (**j**) 5-HIAA, single derivatized, (**k**) 5-HIAL, single derivatized, (**l**) GABA, single derivatized, (**b, h-l**, coronal rat brain section at bregma −2.04 mm). Scale bar, 2 mm. Color scale bars are shown as percentage of maximum intensity. Lateral resolution, 150 µm. Data were normalized against IS, that is, DA-d_4 for DA, DOPAL, DOPAC, 3-MT, MOPAL, 5-HT, NE, DOPEG, 5-HIAL, and 5-HIAA, HVA-d_5 for HVA, and GABA-d_6 for GABA. Groups: FF fresh frozen, n=4; HS heat-stabilized, n=4. The annotation of the brain regions is represented in Figure S5

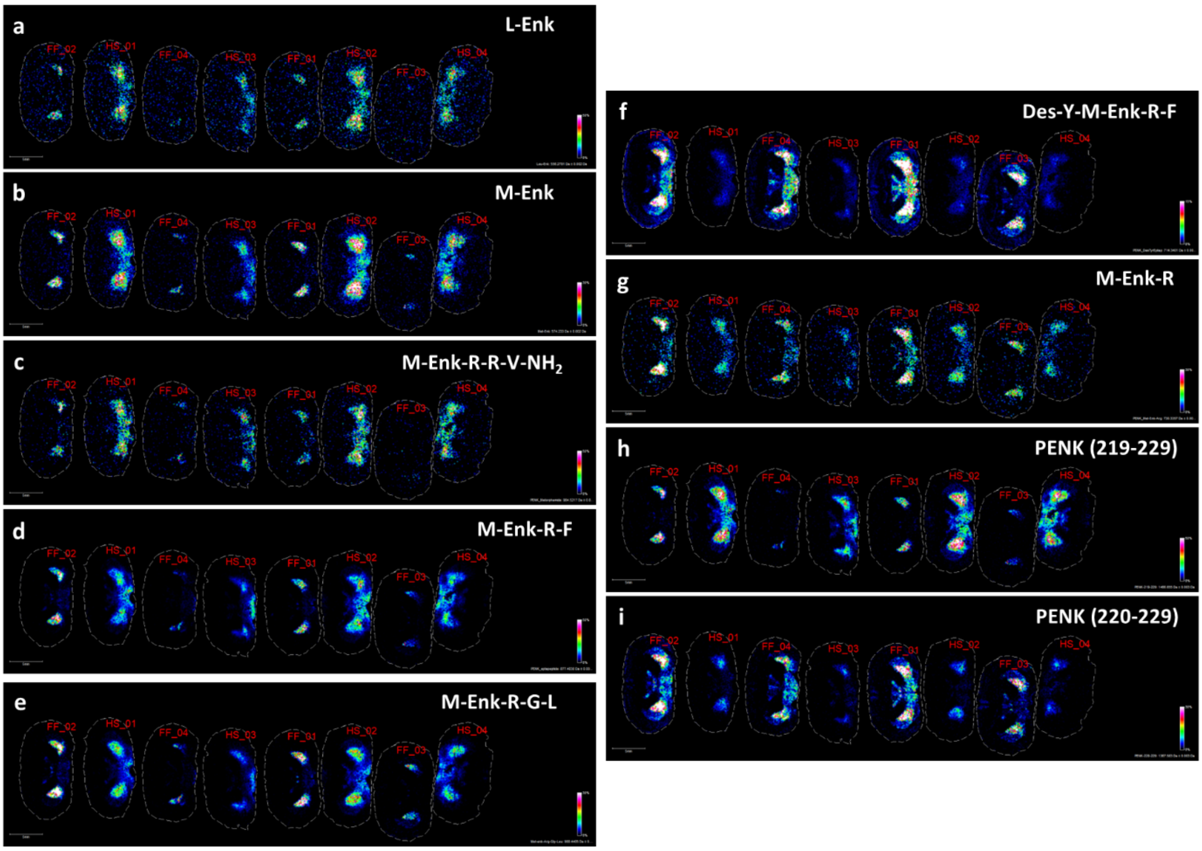

Figure S7. MALDI-MSI images of PENK for the four experimental replicates of the FF and HS samples at bregma -2.04 mm.

(**a**) Leu-Enk, (**b**) Met-Enk, (**c**) Met-Enk-Arg-Arg-Val-NH_2, (**d**) Met-Enk-Arg-Phe, (**e**) Met-Enk-Arg-Gly-Leu, **(f)** Des-Y-Met-Enk-Arg-Phe, (**g)** Met-Enk-Arg, (**h)** PENK (219-229), (**i**) PENK (220-229). Scale bar, 5 mm. Color scale bars are shown as percentage of maximum intensity. Data were normalized against RMS. Lateral resolution, 150 µm. Groups: FF fresh frozen, n=4; HS heat-stabilized, n=4. The annotation of the brain regions is represented in Figure S5.

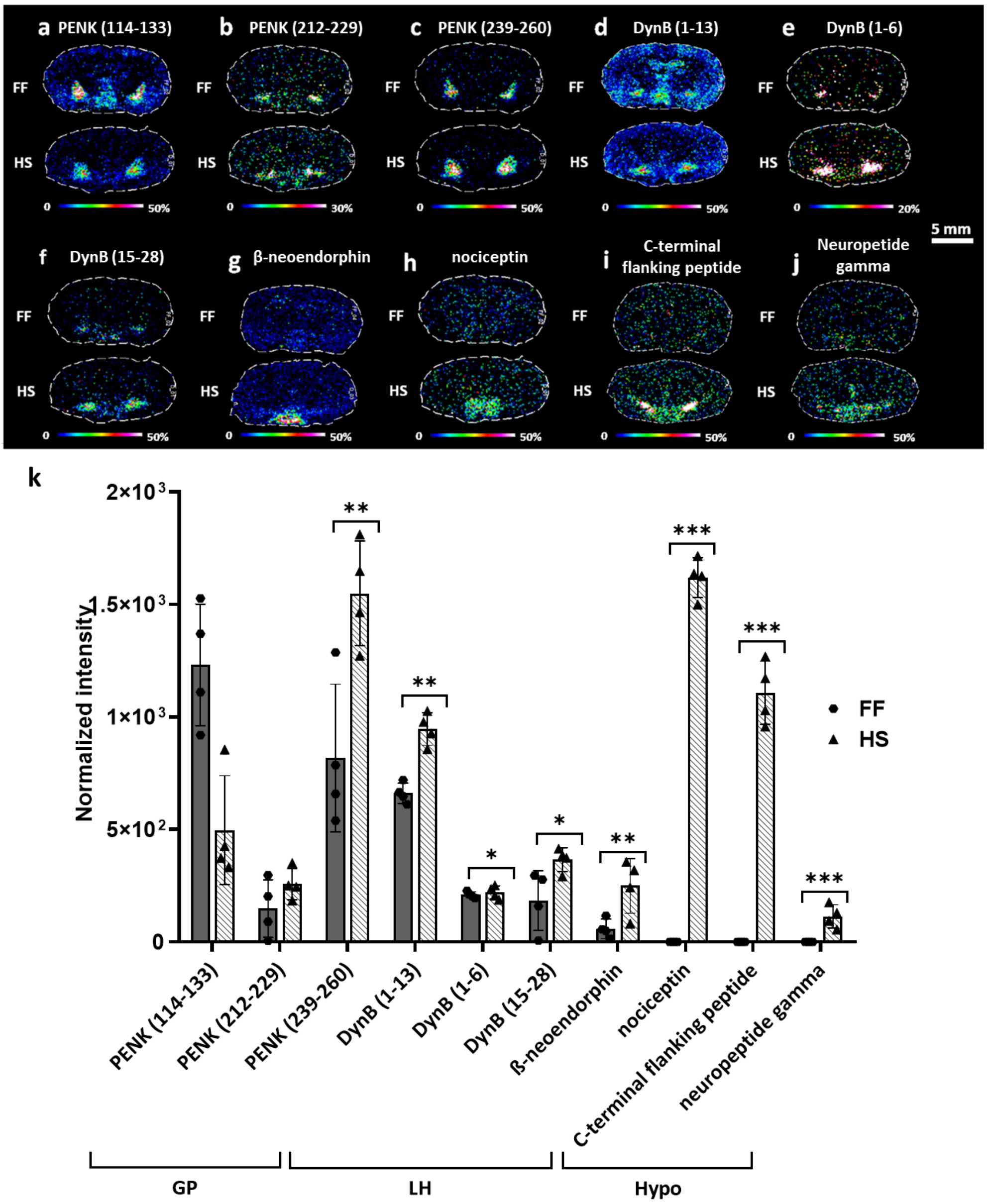

Figure S8. Molecular distributions of PENK (114-133), PENK (212-229), PENK (239-260), DynB (1-13), DynB (1-6), DynB (15-28), β-neoendorphin, nociceptin, C-terminal flanking peptide and neuropeptide-γ determined by MALDI-MSI in coronal rat brain sections comparing fresh frozen with heat-stabilized tissues.

(**a**) PENK (114-133), (**b**) PENK (212-229), (**c**) PENK (239-260), (**d**) DynB (1-13), (**e**) DynB (1-6), (**f**) DynB (15-28), (**g**) β-Neo, (**h**) nociceptin (distance from bregma, -2.04 mm), (**i**) C-terminal flanking peptide, (**j**) neuropeptide-γ (distance from bregma, -2.92 mm), (**k**) Scatter plot reflecting normalized average intensity of full-length peptides and related fragments between globus pallidus (GP), lateral hypothalamus (LH) at bregma -2.04 mm and hypothalamus (Hypo) at bregma -2.92 of HS and FF brain sections. Data are reported as mean ± standard deviation between technical replicates. Statistical analysis was performed by the parametric two-tailed Student's t-test, for independent groups (***$p \leq 0.001$, **$p \leq 0.01$, *$p \leq 0.05$). Scale bar, 5 mm. Colour scale bars are shown as percentage of maximum intensity. Data were normalized against RMS. Lateral resolution, 150 µm. Groups: FF fresh frozen, n=4; HS heat-stabilized, n=4.

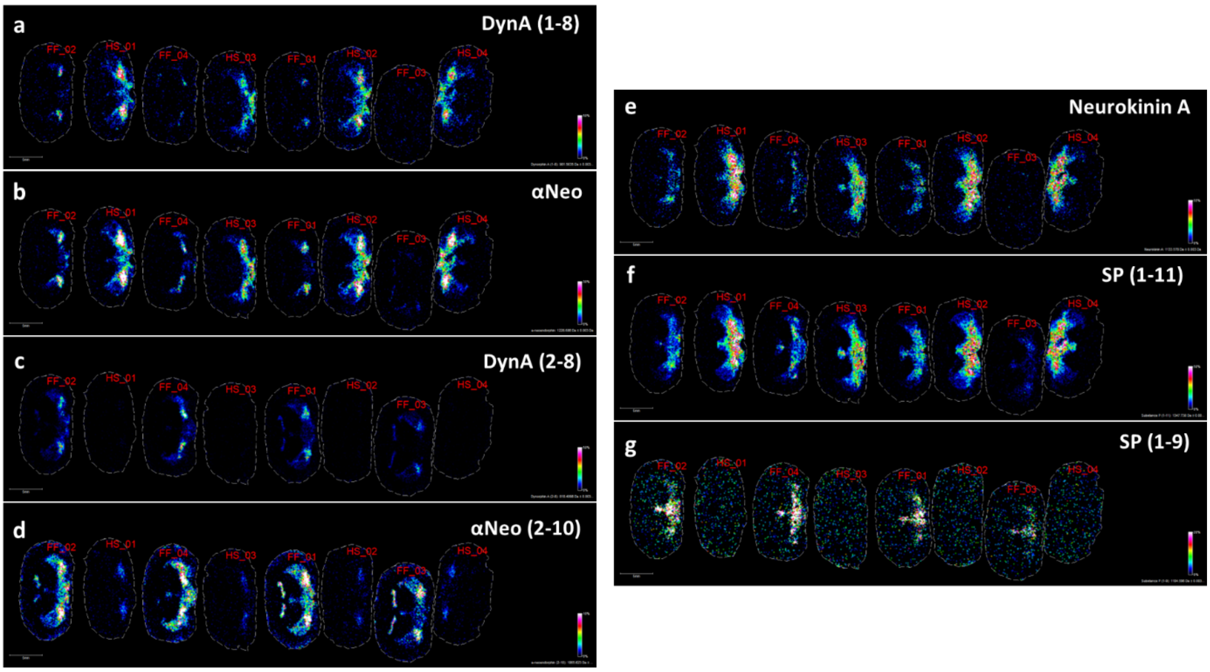

Figure S9. MALDI-MSI images of PDYN and tachykinin for the four experimental replicates of the FF and HS samples at bregma -2.04 mm.

(**a**) DynA (1-8), (**b**) αNeo, (**c**) DynA (2-8), (**d**) αNeo (2-10), (**e**) Neurokinin A, (**f**) SP (1-11), (**g**) SP (1-9). Scale bar, 5 mm. Color scale bars are shown as percentage of maximum intensity. Data were normalized against RMS. Lateral resolution, 150 µm. Groups: FF fresh frozen, n=4; HS heat-stabilized, n=4. The annotation of the brain regions is represented in Figure S5.

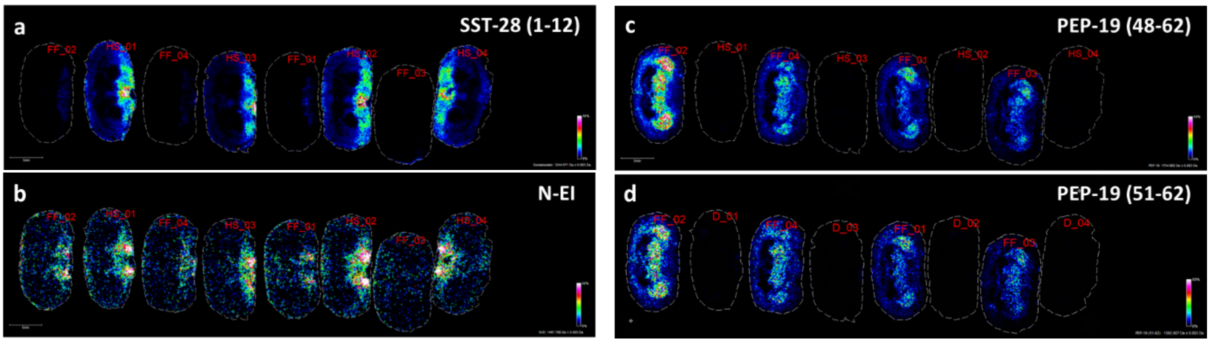

Figure S10. MALDI-MSI images of Somatostatin and PEP-19 for the four experimental replicates of the FF and HS samples at bregma -2.04 mm.

(**a**) SST-28 (1-12), (**b**) N-EI, (**c**) PEP-19 (48-62), (**d**) PEP-19 (51-62). Scale bar, 5 mm. Color scale bars are shown as percentage of maximum intensity. Data were normalized against RMS. Lateral resolution, 150 µm. Groups: FF fresh frozen, n=4; HS heat-stabilized, n=4. The annotation of the brain regions is represented in Figure S5.

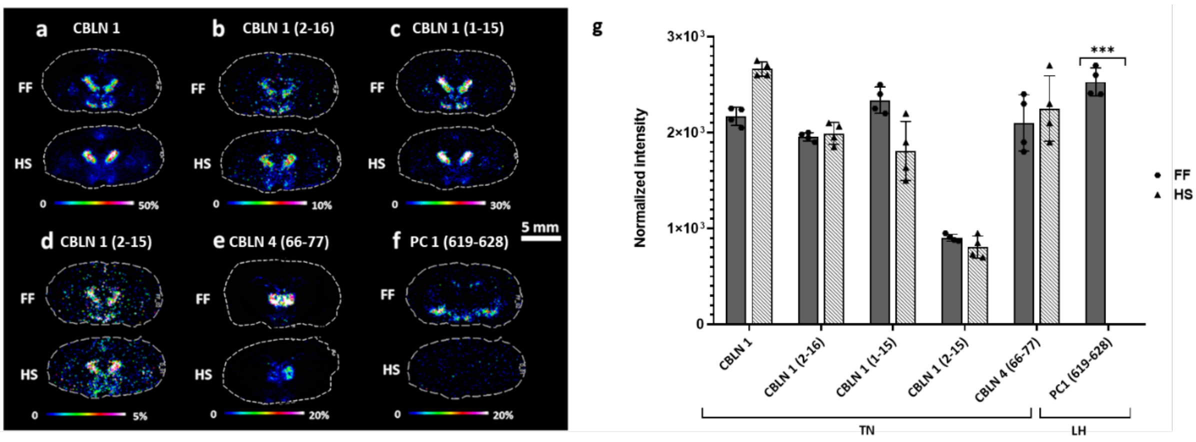

Figure S11. Molecular distributions of cerebellin and prohormone convertase 1 peptides determined by MALDI-MSI in coronal rat brain sections comparing fresh frozen with heat-stabilized tissues.

(**a**) Cerebellin 1, (**b**) cerebellin 1 (2-16), (**c**) cerebellin 1 (1-15), (**d**) cerebellin 1 (2-15), (**e**) cerebellin 4 (66-77), (**f**) PC1 (619-628) (distance from bregma, -2.04 mm), and (**g**) Scatter plot reflecting normalized average intensities of peptides in the thalamic nuclei (TN) and lateral hypothalamus (LH) of HS and FF brain sections. Data are reported as mean ± standard deviation between technical replicates. Statistical analysis was performed by parametric two-tailed Student's t-test, for independent groups (***$p \leq 0.001$). Scale bar, 5 mm. Colour scale bars are shown as percentage of maximum intensity. Data were normalized against RMS. Lateral resolution, 150 µm. Groups: FF fresh frozen, n=4; HS heat-stabilized, n=4. Abbreviations: CBLN, cerebellin; PC1, prohormone convertase 1.

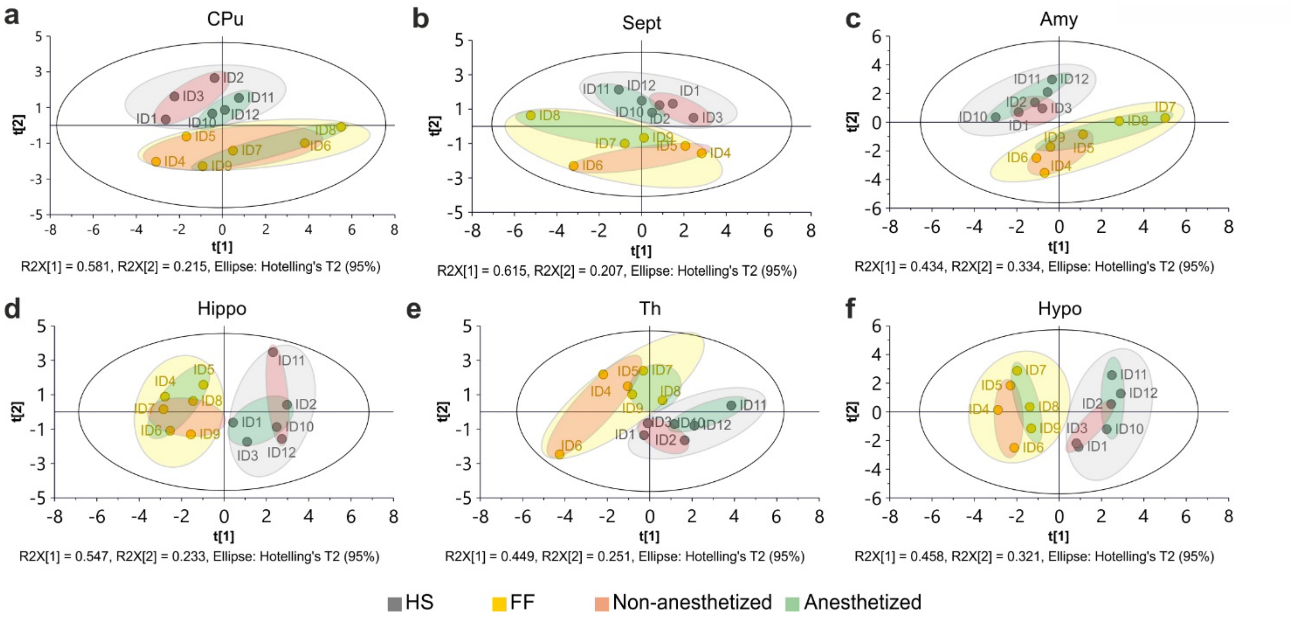

Figure S12. Principal component analysis (PCA) results of MALDI-MSI analysis showing differentiation between fresh frozen and heat-stabilized tissues for brain regions selected for anaesthesia's effect study, with subdivision of anesthetized and non-anesthetized samples.

Data are reported as score plots showing differentiation of HS and FF groups and subgroups in the first and second principal component based on eleven neurotransmitter metabolites from dopaminergic and serotonergic pathway. Abbreviations: FF, fresh frozen; HS, heat-stabilized; CPu, caudate-putamen; Sept, septum; Amy, amygdala; Hippo, hippocampus; Th, thalamus; Hypo, hypothalamus.

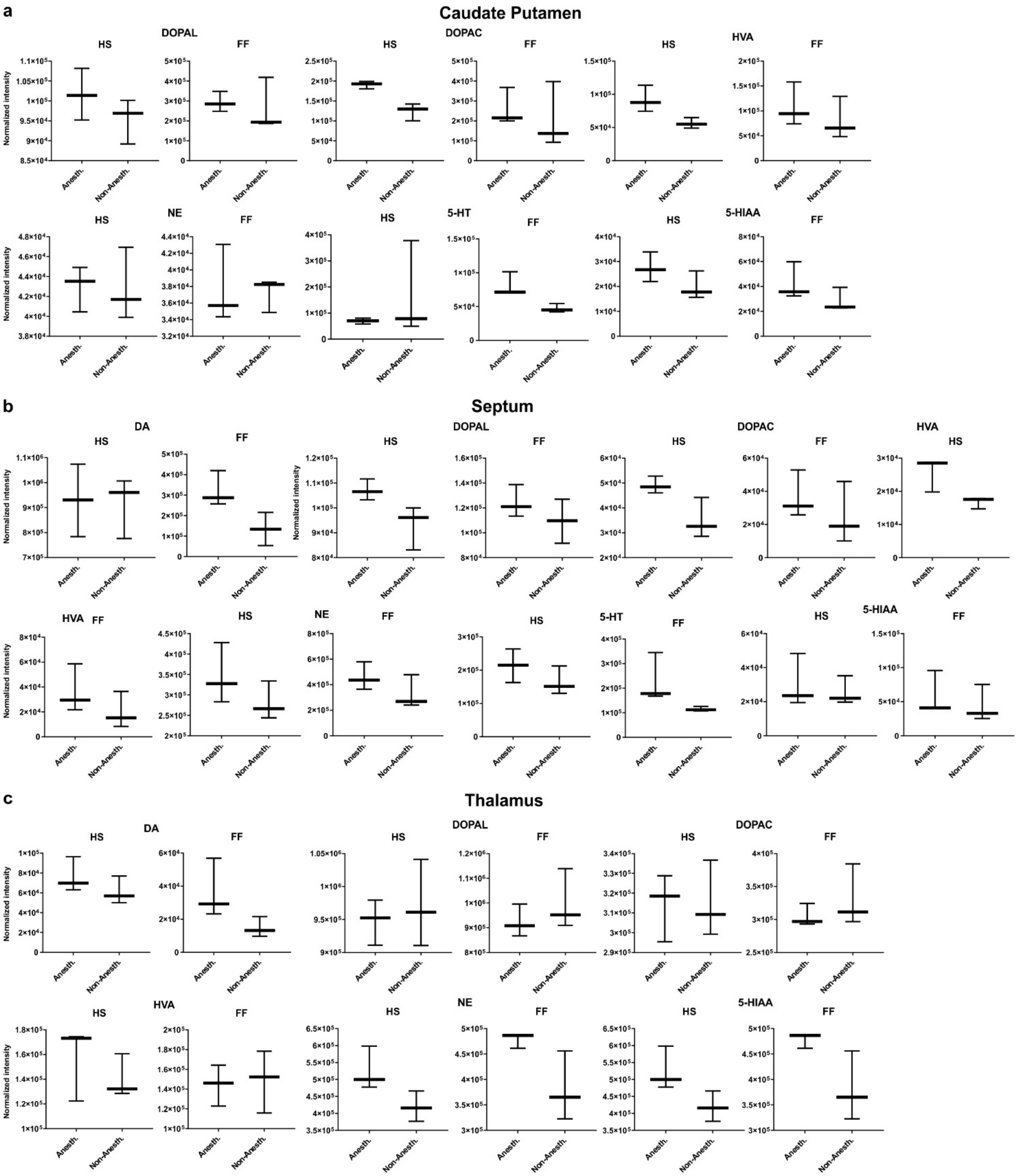

Figure S13. Box plots reflecting normalized average intensity of dopamine and serotonin metabolites in different brain regions.

(a) Caudate-putamen, (b) septum, and (c) thalamus. Data are reported as median with interquartile range. Statistical analysis was performed by nonparametric Mann-Whitney U-test without continuity correction. Abbreviations: FF, fresh frozen; HS, heat stabilized; CPu, caudate-putamen; DA, dopamine; DOPAL, 3,4-dihydroxyphenylacetaldehyde; DOPAC, dihydroxyphenylacetic acid; HVA, homovanillic acid; 5-HT, serotonin; 5-HIAA, 5-hydroxyindoleacetic; NE, norepinephrine; Anesth., anesthetized animals; Non-Anesth, non-anesthetized animals.

Supplementary Tables

Table S1. Neurotransmitters detected in heat-stabilized and fresh frozen brain tissues by MALDI-MSI.

Compound	Theoretical *m/z*	Observed *m/z*	Mass accuracy (ppm)	Internal standard for normalization
DA-double derivatized	674.2802	674.2801	-0.44	DA-d_4 - double derivatized
DOPAL-double derivatized	673.2485	673.2481	-0.43	DA-d_4 - double derivatized
DOPAC-double derivatized	689.2435	689.2434	-1.16	DA-d_4 - double derivatized
3-MT-single derivatized	435.2067	435.2071	0.91	DA-d_4 - single derivatized
MOPAL-single derivatized	434.1745	434.1756	1.38	DA-d_4 - single derivatized
HVA-single derivatized	450.1699	450.1702	0.66	HVA-d_5 - single derivatized
NE-double derivatized	690.2751	690.2755	-0.58	DA-d_4 - double derivatized
DOPEG-double derivatized	691.2591	691.2589	-0.28	DA-d_4 - double derivatized
5-HT-single derivatized	444.2070	444.2074	0.90	DA-d_4 - single derivatized
5-HIAL-single derivatized	443.1754	443.1758	0.91	DA-d_4- single derivatized
5-HIAA single derivatized	459.1703	459.1708	1.08	DA-d_4 - single derivatized
GABA-single derivatized	371.1754	371.1760	1.61	GABA-d_6 - single derivatized

The maximum intensity value of the ion was exported from the average spectrum generated for each brain or annotated brain region by SCiLS Lab and used for statistical analysis. Abbreviations: DA, dopamine; DOPAL, 3,4-dihydroxyphenylacetaldehyde; 3-MT, 3-methoxytyramine; MOPAL, 3-methoxy-4-hydroxyphenylacetaldehyde; HVA, homovanillic acid; NE, norepinephrine; DOPEG, dihydroxyphenyl-ethylene glycol; 5-HT, serotonin; 5-HIAL, 5-hydroxyindoleacetaldehyde; 5-HIAA, 5-hydroxyindoleacetic; GABA, γ-aminobutyric acid.

Table S2. Normalized average intensity with coefficient of variation between biological replicates and fold change of precursors DA, NE and 5-HT and their metabolites between heat-stabilized and fresh frozen protocols. Statistical analysis was performed by parametric two-tailed Student's t-test, for independent groups (p ≤ 0.01, *p ≤ 0.05).**

Bregma 2.28 mm						
**Neurotransmitter**	**DA**	**DOPAL**	**DOPAC**	**MOPAL**	**3-MT**	**HVA**
**Brain tissue**			**Caudate-putamen (CPu)**			
**HS**	6.70E+06	4.04E+05	3.33E+05	1.31E+04	6.94E+04	1.64E+05
**FF**	6.83E+06	6.22E+05	4.82E+05	2.95E+04	8.3E+04	3.49E+05
**CV%**	2.90	28.12	13.01	16.32	10.90	7.44
**Fold change FF/HS**	**1.02**	**1.54 ***	**1.44 ***	**2.25 ****	**1.20**	**2.13 ****
**Fold change HS/FF**	**0.98**	**0.65**	**0.69**	**0.44**	**0.84**	**0.47**

Bregma −2.04 mm						
**Neurotransmitter**	**NE**	**DOPEG**	**5-HT**	**5-HIAA**	**5-HIAL**	**GABA**
**Brain tissue**			**Hypothalamus (Hypo)**			
**HS**	6.19E+05	ND	2.89E+04	1.36E+05	ND	6.04E+05
**FF**	6.24E+05	5.95E+04	3.61E+04	2.94E+05	1.56E+05	8.55E+05
**CV%**	13.99	15.78	7.21	14.23	5.15	7.79
**Fold change FF/HS**	**1.01**	**-**	**1.25**	**2.16 ****	**-**	**1.42 ****
**Fold change HS/FF**	**0.99**	**-**	**0.80**	**0.46**	**-**	**0.71**
**Brain tissue**			**Thalamic nuclei (TN)**			
**HS**	4.77E+05	ND	1.98 E+04	1.13E+05	ND	2.79E+05
**FF**	5.06E+05	7.28E+04	1.55E+04	2.11E+05	1.30E+05	2.94E+05
**CV%**	7.71	10.94	10.37	11.32	8.17	6.69
**Fold change FF/HS**	**1.06**	**-**	**0.78**	**1.87 ****	**-**	**1.05**
**Fold change HS/FF**	**0.94**	**-**	**1.28**	**0.54**	**-**	**0.95**
**Brain tissue**			**Caudate-putamen (CPu)**			
**HS**	-	-	2.03E+04	1.16E+05	ND	3.98E+05
**FF**	-	-	1.58E+04	2.41E+05	1.47E+05	5.62E+05
**CV%**	-	-	18.18	15.68	23.91	16.57
**Fold change FF/HS**	**-**	**-**	**0.78**	**2.08 ****	**-**	**1.41 ****
**Fold change HS/FF**	**-**	**-**	**1.28**	**0.48**	**-**	**0.71**

The maximum intensity value of the ion was exported from the average spectrum generated for each brain region using SCiLS Lab and mean values were used for statistical analysis. Abbreviations: FF, fresh frozen; HS, heat-stabilized; DA, dopamine; DOPAL, 3,4-dihydroxyphenylacetaldehyde; DOPAC, dihydroxyphenylacetic acid; MOPAL, 3-methoxy-4-hydroxy-phenylacetaldehyde; 3-MT, 3-methoxytyramine; HVA, homovanillic acid; NE, norepinephrine; DOPEG, dihydroxyphenylethylene glycol; 5-HT, serotonin; 5-HIAA, 5-hydroxyindoleacetic; 5-HIAL, 5-hydroxyindoleacetaldehyde; GABA, γ-aminobutyric acid; CV, coefficient of variation.

Table S3. Average intensity ratio between metabolite and neurotransmitter for heat-stabilized and fresh frozen brain tissues.

	DOPAL/DA	MOPAL/DA	3-MT/DA	HVA/DA	DOPEG/NE	5-HIAA/5-HT	5-HIAL/5-HT
**Brain tissue**	**Caudate-putamen (CPu)**						
**HS**	0.060 ± 0.10	0.001 ± 0.02	0.010 ± 0.05	0.025 ± 0.01	-	6.670 ± 0.55	2.691± 0.23
**FF**	0.091 ± 0.21	0.008 ± 0.03	0.015 ± 0.02	0.051 ± 0.15	-	13.933 ± 0.78	9.273 ± 0.44
**Brain tissue**	**Hypothalamus (Hypo)**						
**HS**	-	-	-	-	0.015 ± 0.03	5.752 ± 0.60	1.865 ± 0.53
**FF**	-	-	-	-	0.095 ± 0.15	6.556 ± 0.35	4.192 ± 0.24
**Brain tissue**	**Thalamic nuclei (TN)**						
**HS**	-	-	-	-	0.019 ± 0.04	5.191 ± 0.15	2.394 ± 0.47
**FF**	-	-	-	-	0.143 ± 0.13	9.064 ± 0.95	8.344 ± 0.63

Abbreviations: FF, fresh frozen; HS, heat-stabilized; CPu, caudate-putamen; Hypo, hypothalamus; TN, thalamic nuclei; DOPAL, 3,4-dihydroxyphenylacetaldehyde; DA, dopamine; MOPAL, 3-methoxy-4-hydroxyphenylacetaldehyde; 3-MT, 3-methoxytyramine; HVA, homovanillic acid; DOPEG, dihydroxy-phenylethylene glycol; NE, norepinephrine; 5-HIAA, 5-hydroxyindoleacetic; 5-HT, serotonin; 5-HIAL, 5-hydroxyindoleacetaldehyde.

Table S4. Neuropeptides detected in heat-stabilized and fresh frozen brain tissues by MALDI-MSI.

Peptide precursor	Peptide name	Peptide sequence	Theoretical *m/z*	Observed *m/z*	Mass accuracy (ppm)
**Cerebellin 1 (CBLN 1) Precursor protein**	Cerebellin 1 (2-15) #	GSAKVAFSAIRSTN	1408.7543	1408.7570	1.92
	Cerebellin 1 (1-15) #	SGSAKVAFSAIRSTN	1495.7863	1495.7853	-0.66
	Cerebellin 1 (2-16) #	GSAKVAFSAIRSTNH	1545.8132	1545.8129	-0.54
	Cerebellin 1 #	SGSAKVAFSAIRSTNH	1632.8452	1632.8460	0.48
**Cerebellin 4 (CBLN 4)**	Cerebellin 4 (66-77) #	SKVAFSAVRSTN	1266.6800	1266.6828	2.21
**Proenkephalin (PENK)**	Leu-Enk	YGGFL	556.2765	556.2781	1.97
	Met-Enk	YGGFM	574.2329	574.2330	0.01
	Des-Tyr- Met-Enk-Arg-Phe #	GGFMRF	714.3391	714.3401	1.53
	Met-Enk-Arg	YGGFMR	730.3341	730.3357	2.32
	Met-Enk-Arg-Phe (heptapeptide)	YGGFMRF	877.4025	877.4038	1.48
	Met-Enk-Arg-Gly-Leu (octapeptide)	YGGFMRGL	900.4396	900.4405	0.55
	Met-Enk-Arg-Arg-Val-NH_2 (Metorphamide) #	YGGFMRRV-NH_2	984.5196	984.5217	2.03
	PENK (201-209)	LEDEAKELQ	1074.5313	1074.5321	1.02
	PENK (198-207)	SPQLEDEAKE	1145.5320	1145.5340	1.02
	PENK (221-229)	RPEWWMDYQ	1310.5622	1310.5623	0.22
	PENK (220-229)	GRPEWWMDYQ	1367.5837	1367.5830	-0.65
	PENK (198-209)	SPQLEDEAKELQ	1386.6747	1386.6780	2.38
	PENK (219-229)	VGRPEWWMDYQ	1466.6521	1466.6549	1.30
	PENK (114-133) #	MDELYPVEPEEEANGGEILA	2204.9903	2204.9992	4.08
	PENK (212-229) #	YGGFMRRVGRPEWWMDYQ	2334.0695	2334.0765	2.99
	PENK (239-260) #	FAESLPSDEEGESYSKEVPEME	2489.0548	2489.0538	-0.41
**Prodynorphin (PDYN)**	Dynorphin A (1-8)	YGGFLRRI	981.5628	981.5635	0.50
	Dynorphin A (2-8)	GGFLRRI	818.4995	818.4999	-0.12
	Dynorphin A (10-17)	PKLKWDNQ	1028.5523	1028.5540	1.94
	β-neoendorphin #	YGGFLRKYP	1100.5887	1100.5910	1.81
	Dynorphin B (1-13)	YGGFLRRQFKVVT	1570.8852	1570.8822	-1.90
	Dynorphin B (1-6) #	YGGFLR	712.3776	712.3787	1.96
	Dynorphin B (15-28) #	SQENPNTYSEDLDV	1610.6816	1610.6836	1.24
	α-neoendorphin	YGGFLRKYPK	1228.6837	1228.6862	1.55
	α-neoendorphin (3-10)	GFLRKYPK	1008.5989	1008.5979	-0.99
	α-neoendorphin (2-10)	GGFLRKYPK	1065.6203	1065.6230	0.94

**Protachykinin-A**	Neurokinin A	HKTDSFVGLM-NH_2	1133.5771	1133.5778	0.70
	Substance P (1-11)	RPKPQQFFGLM-NH_2	1347.7354	1347.7380	2.25
	Substance P (1-7)	RPKPQQF	900.5050	900.5062	2.22
	Substance P (1-9)	RPKPQQFFG-NH_2	1104.5948	1104.5960	2.22
	C-terminal flanking peptide #	ALNSVAYERSAMQNYE	1845.8435	1845.8410	-1.62
	Neuropeptide-γ #	DAGHGQISHKRHKTDSFVGLM-NH_2	2320.1727	2320.1757	1.29
**Pronociceptin/ Orphanin FQ**	Nociceptin #	FGGFTGARKSARKLANQ	1808.9878	1808.990	1.65
**Prohormone convertase 1 (PC1)**	619-628	GVEKMVNVVE	1103.5765	1103.5770	0.45
**Promelanin concentrating hormone**	Neuropeptide EI #	EIGDEENSAKFPI-NH_2	1447.7063	1447.7062	-0.06
**Proneurotensin**	Neurotensin	QLYENKPRRPYIL	1672.917	1672.9160	-0.59
**Prosomatostatin**	Somatostatin 28 (1-12)	SANSNPAMAPRE	1244.5688	1244.5707	1.60
**Calmodulin regulator protein (PEP-19)**	PEP-19 (48-62)	SQFRKFQKKKAGSQS	1754.9660	1754.9620	-2.27
	PEP-19 (51-62)	RKFQKKKAGSQS	1392.8070	1392.8060	-0.71

neuropeptides detected only in heat-stabilized (HS) brain tissues.

Table S5. Normalized average intensity with coefficient of variation between biological replicates and fold change value of PENK neuropeptides between heat-stabilized and fresh frozen protocols. Statistical analysis was performed by parametric two-tailed Student's t-test, for independent groups (*p ≤ 0.001, **p ≤ 0.01, *p ≤ 0.05).**

Bregma −2.04 mm									
**Peptide**	**Leu-Enk**	**Met-Enk**	**Met-Enk-Arg-Arg-Val-NH_2**	**Met-Enk-Arg-Phe**	**Met-Enk-Arg-Gly-Leu**	**PENK (219-229)**	**PENK Des-Tyr- Met-Enk-Arg-Phe**	**Met-Enk-Arg**	**PENK (220-229)**
**Brain tissue**					**Globus pallidus (GP)**				
**HS**	3.28E+02	2.96E+03	6.62E+02	9.07E+03	8.63E+03	1.46E+04	5.80E+02	1.03E+02	1.78E+02
**FF**	1.17E+02	1.02E+03	2.65E+02	6.98E+03	7.19E+03	3.75E+03	5.68+03	1.96E+02	1.40E+03
**CV%**	19.87	8.08	11.77	10.06	8.56	8.61	10.03	14.24	12.30
**Fold change FF/HS**	**0.36**	**0.34**	**0.40**	**0.77**	**0.83**	**0.26**	**9.80 *** **	**1.91 ** **	**7.85 *** **
**Fold change HS/FF**	**2.81 ***	**2.85 ***	**2.53 ***	**1.31 ***	**1.22 ***	**3.89 ** **	**0.10**	**0.53**	**0.13**
**Brain tissue**					**Caudate-putamen (CPu)**				
**HS**	ND	1.81E+02	5.36E+01	1.00E+03	8.60E+02	1.18E+03	1.09E+01	ND	1.91E+03
**FF**	ND	ND	ND	ND	ND	ND	7.19E+01	ND	8.21E+03
**CV%**	-	6.40	9.05	7.58	9.64	7.32	2.30	-	6.78
**Fold change FF/HS**	-	-	-	-	-	-	**6.60 ** **	-	**4.30 ** **
**Fold change HS/FF**	-	-	-	-	-	-	**0.15**	-	**0.23**
**Brain tissue**					**Lateral hypothalamus (LH)**				
**HS**	ND	ND	ND	5.12E+03	2.45E+03	3.13E+03	2.62E+02	ND	2.09E+03
**FF**	ND	ND	ND	7.18E+02	4.91E+02	ND	1.48E+03	ND	3.21E+03
**CV%**	-	-	-	13.67	8.98	11.68		-	10.98
**Fold change FF/HS**	-	-	-	**0.14**	**0.20**	-	**5.66 ** **	-	**1.53 ***
**Fold change HS/FF**	-	-	-	**7.13**	**5.00**	-	**0.18**	-	**0.65**

Bregma −5.64 mm

Peptide	Met-Enk-Arg-Phe	PENK (219-229)	PENK Des-Tyr- Met-Enk-Arg-Phe	PENK (220-229)
**Brain tissue**	**Periaqueductal gray (PAG)**			
**HS**	7.79E+04	9.49E+04	1.57E+04	2.50E+04
**FF**	3.95E+04	1.59E+04	1.05E+05	1.07E+05
**CV%**	15.11	4.40	5.21	17.89
**Fold change FF/HS**	**0.51**	**0.17**	**6.67 ****	**4.28 ***
**Fold change HS/FF**	**1.97 ***	**5.97 *****	**0.15**	**0.23**

Brain tissue	Substantia nigra (SN)			
**HS**	2.45E+04	2.58E+04	4.33E+03	6.03E+03
**FF**	9.21E+03	4.12E+03	3.23E+04	2.60E+04
**CV%**	11.15	9.33	7.08	20.05
**Fold change FF/HS**	**0.38**	**0.16**	**7.47 ****	**4.31 ****
**Fold change HS/FF**	**2.66 ***	**6.25 *****	**0.13**	**0.23**

Bregma −2.92 mm		
**Peptide**	**PENK (198-209)**	**PENK (201-209)**
**Brain Tissue**	**Amygdala (Amy)**	
**HS**	2.91E+04	ND
**FF**	2.01E+04	1.30E+03
**CV%**	6.31	5.25
**Fold change FF/HS**	**0.69**	-
**Fold change HS/FF**	**1.45 ***	-

The maximum intensity value of the ion was exported from the average spectrum generated for each brain region using SCiLS Lab and mean values were used for statistical analysis. Abbreviations: FF, fresh frozen; HS, heat-stabilized; CV, coefficient of variation.

Table S6. Normalized average intensity with coefficient of variation between biological replicates and fold change value of PDYN and tachykinin neuropeptides between HS and FF protocols. Statistical analysis was performed by parametric two-tailed Student's t-test, for independent groups (*p ≤ 0.001, **p ≤ 0.01, *p ≤ 0.05).**

**Bregma −2.04 mm**							
**Peptide**	**DynA (1-8)**	**α-Neo**	**DynA (2-8)**	**αNeo (2-10)**	**Neurokinin A**	**SP (1-11)**	**SP (1-9)**
**Brain tissue**	**Lateral hypothalamus (LH)**						
**HS**	1.88E+03	5.92E+03	3.01E+02	3.20E+02	2.07E+03	8.19E+03	ND
**FF**	4.87E+02	2.57E+03	2.97E+03	3.84E+03	5.21E+02	3.59E+03	1.32E+02
**CV%**	6.63	6.80	22.39	12.48	10.75	6.30	12.25
**Fold change FF/HS**	**0.26**	**0.43**	**9.89 ****	**12.01 *****	**0.25**	**0.44**	**-**
**Fold change HS/FF**	**3.86 ****	**2.30 ****	**0.10**	**0.08**	**3.97 *****	**2.28**	**-**
**Bregma −5.64 mm**							
**Brain tissue**	**Periaqueductal gray (PAG)**						
**HS**	1.42E+04	4.40E+04	1.39E+03	2.93E+03	7.43E+04	3.10E+05	ND
**FF**	3.76E+03	2.13E+04	1.98E+04	4.05E+04	3.52E+04	1.46E+05	5.02E+04
**CV%**	5.97	20.40	8.08	18.83	5.68	2.52	8.23
**Fold change FF/HS**	**0.26**	**0.48**	**14.18 ***	**13.82 ***	**0.47**	**0.45**	**-**
**Fold change HS/FF**	**3.78 ****	**2.06 ***	**0.07**	**0.07**	**2.11 ****	**2.12 ****	**-**
**Brain tissue**	**Substantia nigra (SN)**						
**HS**	1.83E+05	4.14E+05	8.82E+03	3.25E+04	1.69E+05	4.42E+05	ND
**FF**	4.94E+04	1.84E+05	8.90E+04	3.67E+05	6.46E+04	2.46E+05	3.38E+04
**CV%**	12.40	9.67	12.34	6.18	7.22	9.20	15.80
**Fold change FF/HS**	**0.27**	**0.44**	**10.09 ****	**11.31 ****	**0.38**	**0.56**	**-**
**Fold change HS/FF**	**3.70 ***	**2.25 ***	**0.10**	**0.09**	**2.61 ****	**1.80 ***	**-**

The maximum intensity value of the ion was exported from the average spectrum generated for each brain region using SCiLS Lab and mean values were used for statistical analysis. Abbreviations: FF, fresh frozen; HS, heat-stabilized; CV, coefficient of variation.

Table S7. Normalized average intensity with coefficient of variation between biological replicates and fold change value of Somatostatin 28 (1-12) and Neuropeptide EI between heat-stabilized and fresh frozen protocols. Statistical analysis was performed by parametric two-tailed Student's t-test, for independent groups (*p ≤ 0.001, **p ≤ 0.01, *p ≤ 0.05).**

Bregma −2.04 mm		
**Peptide**	**SST-28 (1-12)**	**N-EI**
**Brain Tissue**	**Hypothalamus (Hypo)**	
**HS**	1.21E+05	9.99E+03
**FF**	8.74E+03	6.54E+03
**CV%**	12.41	11.71
**Fold change FF/HS**	**0.07**	**0.65**
**Fold change HS/FF**	**13.80 *** **	**1.53**

The maximum intensity value of the ion was exported from the average spectrum generated for each brain region using SCiLS Lab and mean values were used for statistical analysis. Abbreviations: FF, fresh frozen; HS, heat-stabilized; CV, coefficient of variation.

Reference

Paxinos, G. and Watson, C. (2014) *Paxino's and Watson's The rat brain in stereotaxic coordinates*. Elsevier/AP, Academic Press is an imprint of Elsevier, Amsterdam; Boston.
